# Senolytic reduction of senescent cells mitigates atrial arrhythmia vulnerability in aging rabbits

**DOI:** 10.1016/j.hrthm.2026.01.007

**Published:** 2026-01-07

**Authors:** Elif Sengun, Lily Zhou, Maxfield Kelsey, Nilufer N. Turan, Yichun Lu, Anatoli Y. Kabakov, Peter Bronk, Eric Mi, Tae Yun Kim, Shilpa Vijayakumar, Dana Price, Sade Solola Nussbaum, Christopher Song, Jun Feng, Frank W. Sellke, Patrycja Dubielecka-Szczerba, Federica Del Monte, Jeanne Nerbonne, B. Sonmez Uydes-Dogan, Karim Roder, Bum-Rak Choi, David R. Van Wagoner, John M. Sedivy, Gideon Koren

**Affiliations:** 1 The Warren Alpert Medical School of Brown University, Providence, Rhode Island; 2 Cardiovascular Research Center, Rhode Island Hospital, Providence, Rhode Island; 3 Department of Pharmacology, Institute of Graduate Studies in Health Sciences, Istanbul University, Istanbul, Tuürkiye; 4 Department of Molecular Biology, Cell Biology and Biochemistry, Brown University, Providence, Rhode Island; 5 School of Nursing and Health Sciences, Providence College, Providence, Rhode Island; 6 Department of Hematology & Oncology, Rhode Island Hospital, Providence, Rhode Island; 7 Medical University of South Carolina, Charleston, South Carolina; 8 Department of Developmental Biology, Medical School of Washington University, St. Louis, Missouri; 9 Department of Pharmacology, Faculty of Pharmacy, Istanbul University, Istanbul, Tuürkiye; 10 Department of Heart, Blood and Kidney Research, Cleveland Clinic, Cleveland, Ohio.

**Keywords:** Atrial fibrillation, Cellular senescence, Aging, Senescence-associated secretory phenotype (SASP), Inflammation, Atrial remodeling, Senolytic therapy

## Abstract

**BACKGROUND:**

Atrial fibrillation (AF) is the most common arrhythmia among the elderly and a major contributor to morbidity and mortality. Inflammation plays a central role in AF pathogenesis, and aging is a key independent risk factor. Cellular senescence is a hallmark of aging and contributes to age-related disease through the senescence-associated secretory phenotype (SASP), characterized by proinflammatory and profibrotic factors.

**OBJECTIVE:**

This study aimed to determine whether senescent atrial cells contribute to age-related AF risk and whether senolytic therapy can mitigate this phenotype.

**METHODS:**

Young (≤1 year) and aged (≥4 years) New Zealand White rabbits were evaluated using optical mapping, patch-clamp electrophysiology, and histologic and molecular analyses. Senescence markers were assessed using senescence-associated β-galactosidase staining, immunofluorescence, and RNA sequencing. Human atrial specimens from patients with and without AF were examined to assess translational relevance. Aged rabbits received the senolytic compound fisetin to evaluate its effects on atrial senescence and arrhythmia susceptibility.

**RESULTS:**

Aged rabbits displayed electrophysiological heterogeneity, prolonged action potentials, and increased AF inducibility, recapitulating clinical features of elderly human atria. Atrial tissue from aged rabbits and patients with AF showed an increase in senescent myocytes and myofibroblasts with upregulation of inflammatory SASP genes. SASP factor expression correlated with left atrial diameter in human samples, an AF risk factor. Short-term fisetin treatment eliminated most senescent atrial cells, reduced inducible AF, and decreased reentry activity without impairing atrial function.

**CONCLUSION:**

Senescent atrial cells promote a proinflammatory, proarrhythmic substrate predisposing to AF. Senolytic therapy with fisetin alleviates this phenotype, suggesting a potential strategy to prevent age-related AF.

Atrial fibrillation (AF) is the most prevalent arrhythmia among the elderly, with a lifetime risk of 25% after the age of 40 years^1^ and an anticipated prevalence exceeding 12 million individuals in the United States by 2030.^2^ Its clinical significance lies not only in its rising incidence but also in its association with severe complications, including stroke, heart failure, and increased mortality.^3,4^ Although aging is the strongest independent risk factor for AF,^5,6^ the biological processes linking aging to atrial vulnerability remain incompletely understood.

One biological process closely tied to aging is cellular senescence, a state of stable cell-cycle arrest triggered by diverse stressors such as DNA damage, oxidative stress, and metabolic imbalance.^7,8^ Senescent cells remain metabolically active and secrete a complex mix of cytokines, chemokines, and proteases, collectively termed the senescence-associated secretory phenotype (SASP). The SASP promotes inflammation, extracellular matrix remodeling, and electrical instability—features that overlap with the known drivers of AF pathogenesis.^9^

Despite growing evidence linking cellular senescence to age-associated cardiac remodeling, the contribution of senescence to electrophysiological alterations that predispose to arrhythmias remains unclear. In particular, senescence-driven structural and molecular changes that influence atrial conduction, repolarization, and arrhythmogenic vulnerability have not been systematically investigated in a translational large-animal model. Most previous studies have relied on rodent systems, which differ substantially from humans in atrial size, ion-channel composition, and electrophysiological properties. Here, we address this critical knowledge gap by integrating electrophysiological, histologic, and molecular analyses in an established rabbit model of cardiac aging, which we have previously shown effectively recapitulates the electrophysiological features of the aged human heart,^10^ while also evaluating the novel impact of pharmacologic senolytic intervention. This comprehensive approach enables direct examination of the mechanistic interface among aging, cellular senescence, and atrial electrophysiology and allows exploration of a potential therapeutic strategy to mitigate age-related atrial vulnerability.

## Methods

### Animal ethical statement

This study was approved by the Rhode Island Hospital Institutional Animal Care and Use Committee (CMTT #5034-21) and adhered to the American Physiological Society’s Guiding Principles for Research Involving Animals and Human Beings and the National Institutes of Health guide for the Care and Use of Laboratory Animals (National Institutes of Health publication, revised 2011).

### Human ethical statement

The Institutional Review Board of Rhode Island Hospital has reviewed and approved Dr Gideon Koren’s human studies (project 1362230-4, renewal on August 9, 2023). He is exempt from the secondary uses of identifiable private information or identifiable biospecimens. A summary of available patient characteristics, including sex, rhythm status at the time of surgery, and available clinical metadata, is presented in Supplemental Table 1.

### Data availability statement

To prevent potential misinterpretation of the raw data and ensure proper attribution, the data and study materials that support the findings of this study can be obtained upon reasonable request from the corresponding author.

Details about all experimental procedures, protocols, and statistical analyses are presented in the Supplemental Material.

## Results

### Aged rabbit atria are more prone to arrhythmias

**Figure 1:**
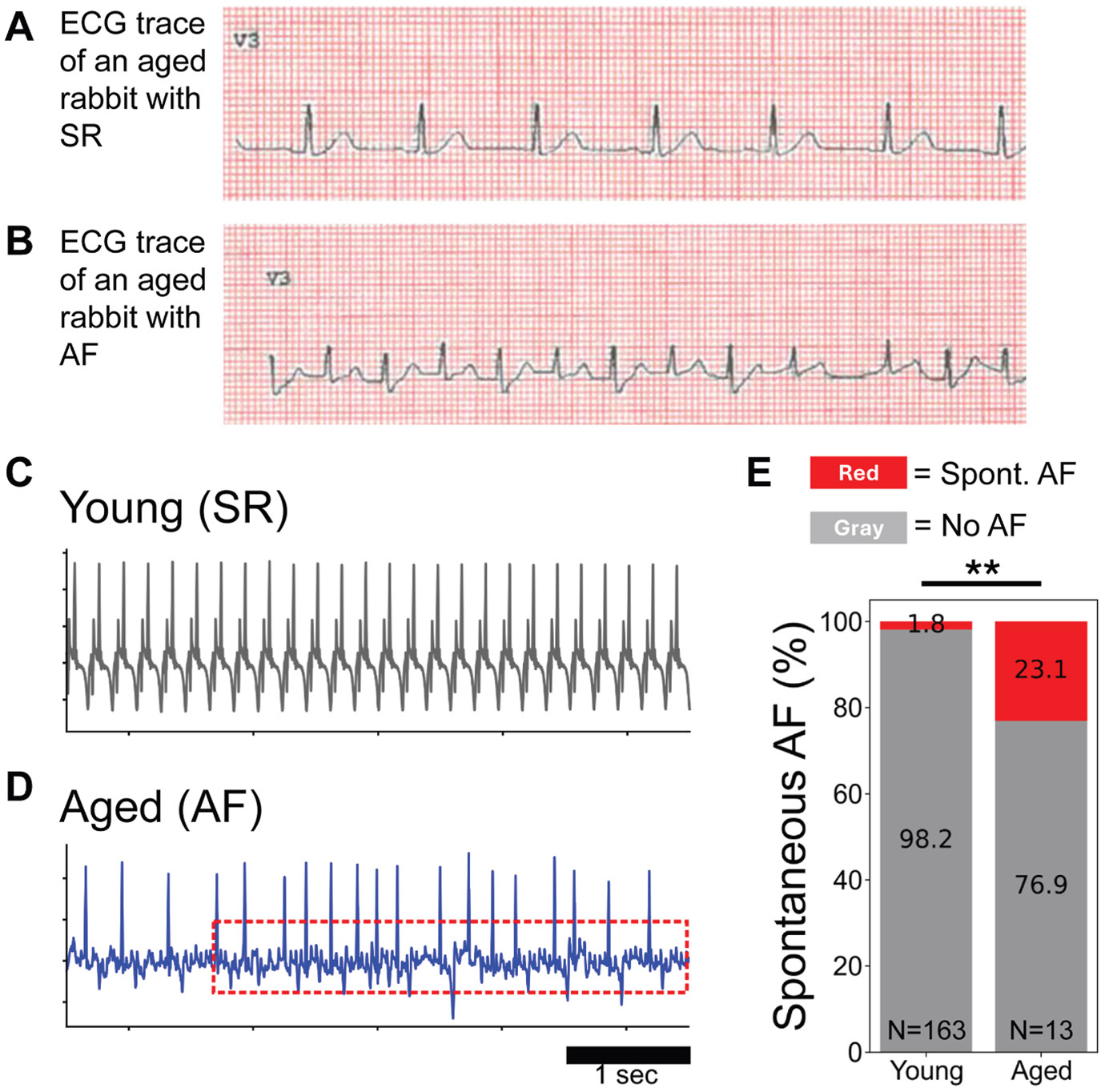
Spontaneous atrial fibrillation in an aged rabbit heart. **A:** Sample in vivo ECG trace of sinus rhythm in an aged rabbit. **B:** Sample in vivo ECG trace of atrial fibrillation in an aged rabbit. Spontaneous AF episodes were identified based on irregular R-R intervals, absence of discernible P waves, and rapid atrial activity consistent with AF. ECGs were recorded at 50 mm/s. **C:** Sample ex vivo ECG traces showing normal sinus rhythm in a representative Langendorff-perfused young rabbit heart. **D:** A representative trace of spontaneous AF that occurred and persisted during Langendorff perfusion in a subset of aged rabbit hearts. **E:** Historic data of ex vivo intact heart optical mapping show that 23.1% of aged rabbit atria showed spontaneous AF (*red*) (n = 3/13), whereas young atria showed spontaneous AF (*red*) rarely (n = 3/163; 1.8%) compared with no spontaneous AF (*gray*). Fisher’s exact test **P < .01. AF = atrial fibrillation; ECG = electrocardiogram; SR = sinus rhythm.

We first studied the electrophysiological characteristics of young and aged rabbit atria and investigated whether AF in aged rabbit hearts replicated important clinical features of AF in the aged human population. Electrocardiography (ECG) and echocardiography recordings on all rabbits in our colony were used to create a database and verify the spontaneous AF incidence in the aged rabbit cohort (Figure 1A and 1B). Spontaneous AF developed in 4 of 32 aged rabbits. In 2 cases, spontaneous AF persisted throughout the ECG and echocardiogram recordings and continued during subsequent optical mapping (OM) experiments performed for a total duration of 6–7 hours, indicating a sustained arrhythmic phenotype. In the ex vivo OM setting, spontaneous AF episodes lasted 393.5 ± 128.1 seconds on average (n = 5 episodes in 3 of 13 hearts). These data demonstrate prolonged spontaneous AF phenotypes in aged rabbit atria (Figure 1C-1E).

**Figure 2:**
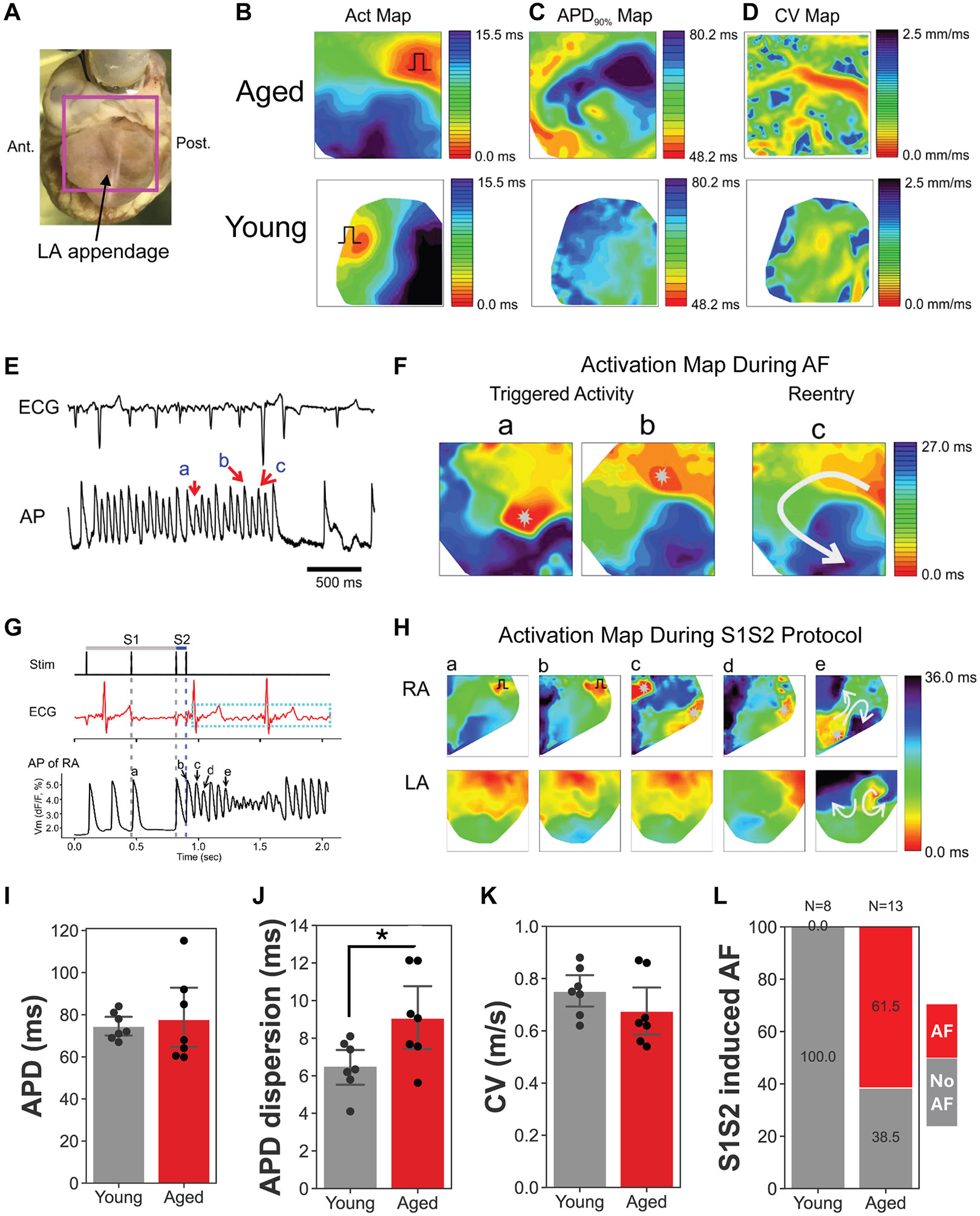
Characterization of atrial action potential and mapping of reentry during AF in the aged rabbit heart. **A:** Optical mapping region of aged atrial tissue. Hearts were excised from the chest of euthanized rabbits and perfused in a Langendorff perfusion apparatus. Hearts were stained with the voltage-sensitive dye di-4-ANEPPS. The pacing electrode was placed on the sinoatrial nodal region to test vulnerability to AF under stimulation protocol. **B–D:** Activation, action potential duration, and conduction velocity maps of young and aged atria during sinus rhythm. These maps show a spatially heterogeneous APD pattern with nonuniform conduction in aged atrial tissue. Location of pacing electrode indicated by the square pulse drawn on activation maps. **E:** Sample ECG and atrial action potential traces during spontaneous AF in an aged rabbit. Letters above the AP trace (bottom panel) indicate the point in time represented by the activation maps in panel F. **F:** Action potential activation map during AF in aged rabbit atrial tissue. Both focal (maps Fa and b, *gray stars*) and reentry (map Fc) underlie maintenance of AF. **G:** S1S2 stimulation protocol (top panel, cycle length: S1 = 350 ms, S2 = 80 ms) on the right atria performed to test AF inducibility in the rabbit heart. The *blue dashed rectangle* indicates atrial fibrillation on the ECG trace. Letters above the AP trace (bottom panel) indicate the point in time represented by the APD maps in panel H. **H:** Both reentry and multiple focal activity play an important role in maintaining the AF induced by the S1S2 stimulation protocol. Location of pacing electrode indicated by the square pulse drawn on maps a and b. Cycle length: S1 = 350 ms, S2 = 80 ms, see panel G (Stim.). **I–K:** APD measured at APD_90_, APD dispersion, and CV in young (*gray*) vs aged (*red*) rabbit atria. There are trends in CV reduction (*P* = .24) and APD prolongation (*P* = .39) and significantly increased APD dispersion (*P* = .03) in the aged rabbit left atria (n = 7). **L:** AF (*red*) was successfully induced with the S1S2 stimulation protocol in 61.5% of aged rabbits (n = 8/13), whereas no instances of induced AF (gray) (n = 0/8) were observed in young rabbits. AF = atrial fibrillation; AP = action potential; APD = action potential duration; APD_90_ = APD at 90% repolarization level; CV = conduction velocity; ECG = electrocardiogram; LA = left atrium; RA = right atrium.

OM was used to study AF vulnerability in aged hearts. Maps of activation, action potential duration (APD), and conduction velocity (CV) from aged atria (Figure 2A-2D) showed a large APD dispersion between posterior and anterior regions (Figure 2C) and CV heterogeneity (Figure 2D) near the pulmonary vein (right top quadrant of CV map). Activation maps of a short spontaneous AF episode in Figure 2E and 2F show that both multiple focal activity (maps “a” and “b”) and rotors (map “c”) maintain AF similar to AF in humans.^11^ S1S2 protocol–induced AF had an average duration of 7.2 ± 7.8 seconds (n = 11 episodes in 8 of 13 aged hearts) (Figure 2G) but not in young atria (n = 0/8). Detailed maps of AF showed that both focal activity and reentry in the right atrium (RA) and left atrium (LA) maintain AF in these aged hearts (Figure 2H). Of note, AF was inducible in 8 of 13 aged rabbit hearts using pacing, suggesting that aged rabbit atria have a low threshold for evoking atrial arrhythmias. Importantly, none of the 8 young rabbits were inducible by pacing. Data of CV, APD, APD dispersion, and AF inducibility under S1S2 programmed stimulation of young and aged hearts are presented in Figure 2I-2L. APD dispersion was significantly higher in the aged atria (*P* = .03), explaining frequent reentry and AF induction under the S1S2 stimulation protocol. However, slowing of CV reduction (*P* = .24) and APD prolongation (*P* = .39) did not reach statistical significance owing to increased intragroup variation in aged rabbits (standard deviation [SD] of CV 0.08 m/s in young vs 0.12 m/s in old; SD of APD 6 ms in young vs 19 ms in aged).

### Electrophysiological remodeling in aging atrial myocytes

**Figure 3:**
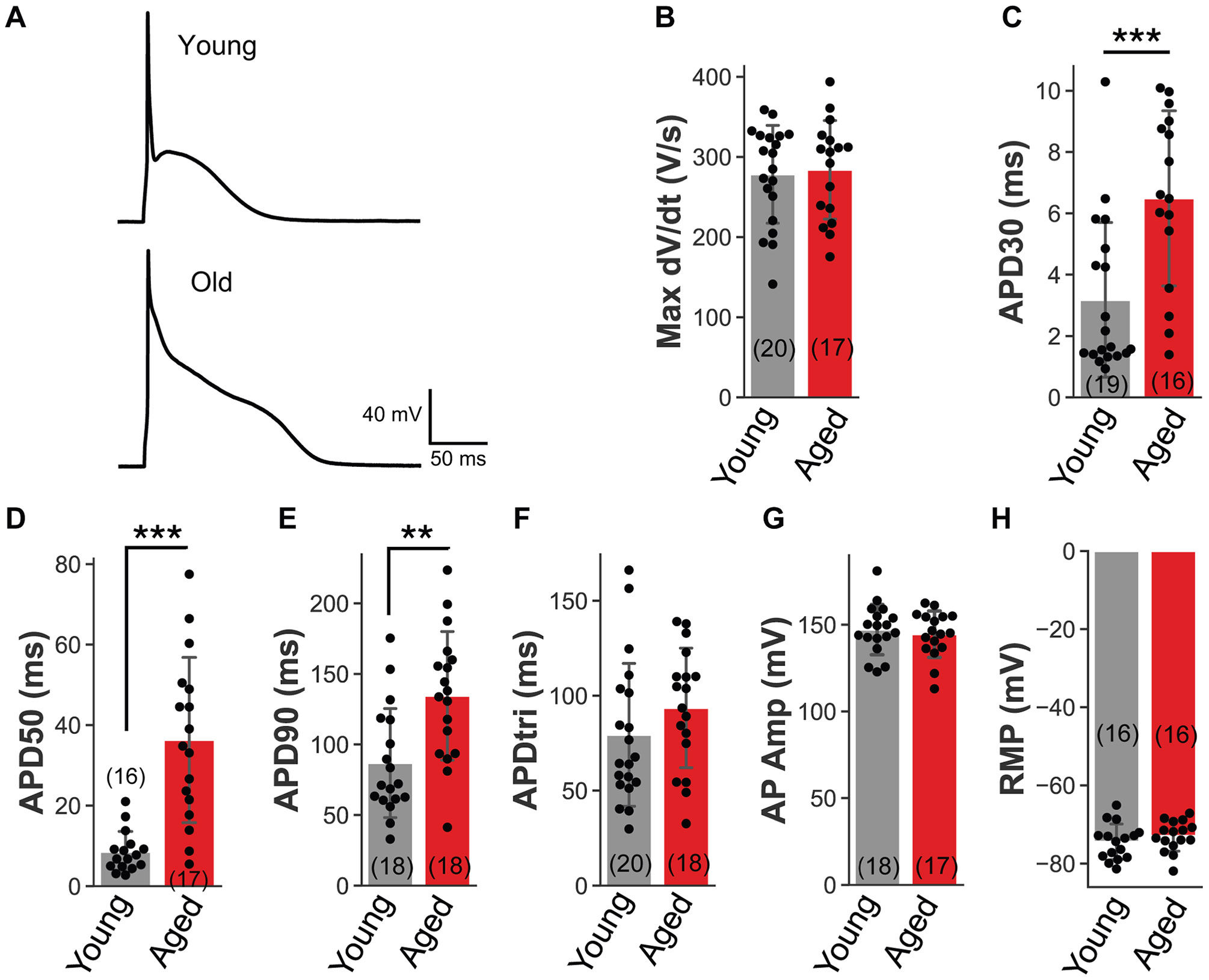
APs of atrial myocytes from young and aged rabbits. **A:** Representative AP waveforms of freshly isolated atrial myocytes from young and aged rabbits stimulated at 1.25 Hz. Adult atrial myocytes were isolated via Langendorff perfusion and enzymatic digestion. Whole-cell patch-clamp recordings were performed at 35°C–37°C. APs were evoked in current clamp mode by 3 ms depolarizing pulses at 1.25 Hz. The voltage output was filtered by a low-pass filter with a cutoff frequency of 10 kHz and sampled at 20 kHz. AP parameters were calculated using Python 3.11. **B:** Mean maximum slope of AP rise (dV/dt) in young (19 cells, 4 hearts) and old rabbit atrial myocytes (16 cells, 4 hearts). Aged maximum dV/dt is not changed compared with young (*t* test, equal variance, *P* = .79). **C:** Mean APD at 30% repolarization (APD_30_) of young (19 cells, 4 hearts) and old rabbit atrial myocytes (16 cells, 4 hearts). Aged APD_30_ is significantly longer than young APD_30_ (*t* test, equal variance, *P* = .0009). **D:** Mean APD at 50% repolarization (APD_50_) of young (16 cells, 4 hearts) and old rabbit atrial myocytes (17 cells, 4 hearts). Aged APD_50_ is significantly longer than young APD_50_ (*t* test, unequal variance, *P* = 3.8e-5). **E:** Mean APD at 90% repolarization (APD_90_) of young (18 cells, 4 hearts) and old rabbit atrial myocytes (18 cells, 4 hearts). Aged APD_90_ is significantly longer than young APD_90_ (*t* test, equal variance, *P* = .002). **F:** Mean APD triangulation (APDtri = APD_90_ – APD_50_) of young (20 cells, 4 hearts) and old rabbit atrial myocytes (18 cells, 4 hearts). No significant difference (*t* test, equal variance, *P* = .2). **G:** Mean AP amplitude of young (18 cells, 4 hearts) and old rabbit atrial myocytes (17 cells, 4 hearts). No significant difference (*t* test, equal variance, *P* = .6). **H:** Mean resting membrane potential (RMP) of young (16 cells, 4 hearts) and old rabbit atrial myocytes (16 cells, 4 hearts). No significant difference (*t* test, equal variance, *P* = .3). All column plots show mean ± standard deviation. Scatter points represent individual atrial myocytes (number of cells in parentheses). AP = action potential; APD = action potential duration.

Patch-clamp recordings of atrial myocytes freshly isolated from aged and young rabbits showed that APD at 30% repolarization level (young 3.2 ± 2.5 ms; aged 6.5 ± 2.7 ms; *P* < .001), APD at 50% repolarization level (young 8.5 ± 4.9 ms; aged 36.3 ± 19.9 ms; *P* < .001), and APD at 90% repolarization level (APD_90_) (young 86.8 ± 37.5 ms; aged 134.4 ± 44.3 ms; *P* < .01) were significantly prolonged in aged atrial myocytes compared with young (Figure 3). There were no significant differences in maximum dV/dt (young 278.3 ± 59.5 V/s; aged 283.9 ± 59.7 V/s), action potential amplitude (young 147.2 ± 14.1 mV; aged 144.6 ± 13 mV), action potential triangulation (young 79.4 ± 36.7 ms; aged 93.5 ± 30.6 ms), or resting membrane potential (young −74.4 ± 4.4 mV; aged −72.9 ± 3.8 mV).

### Elevated senescent cell burden across specific cell types in aged rabbit atria

**Figure 4:**
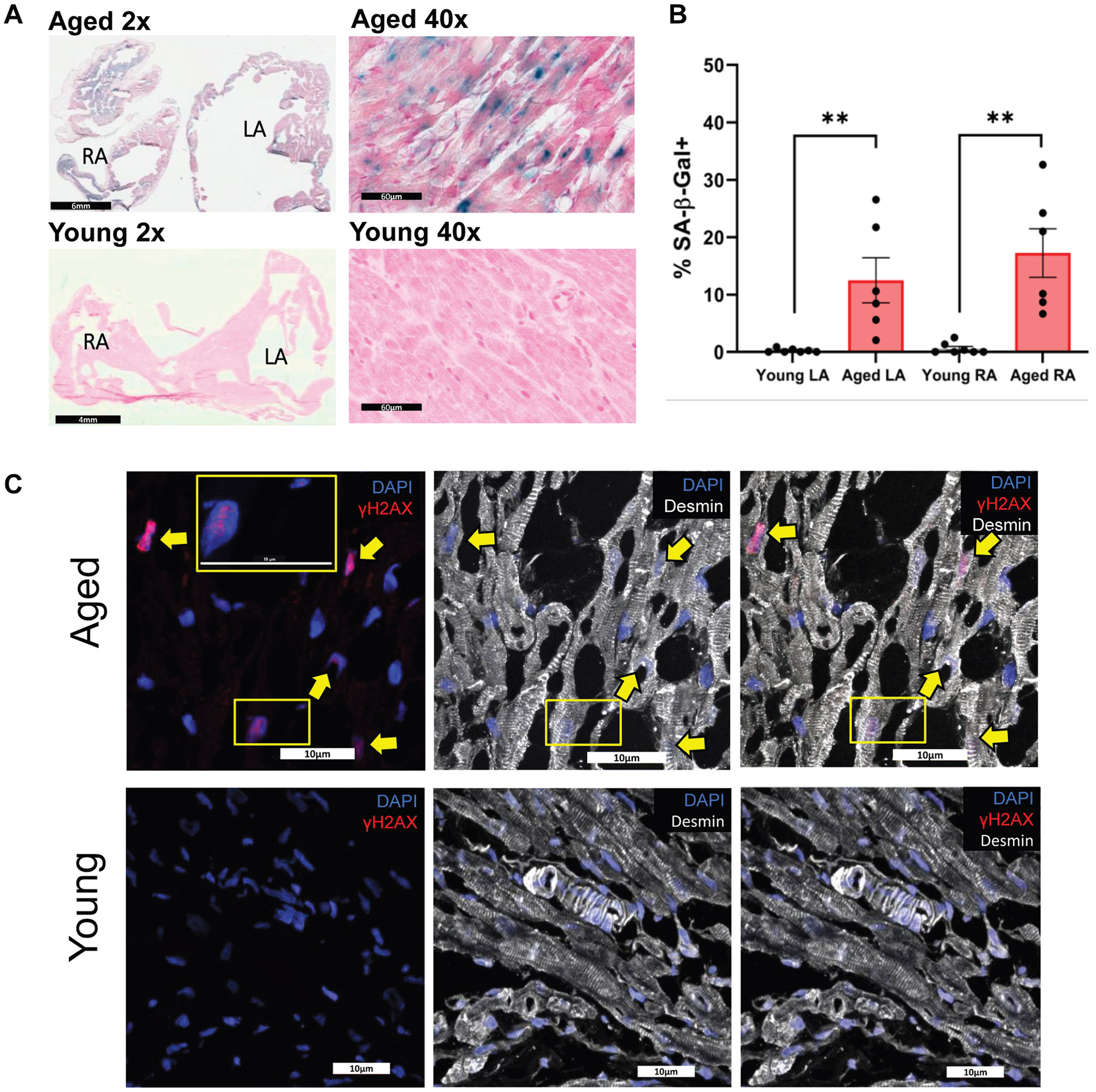

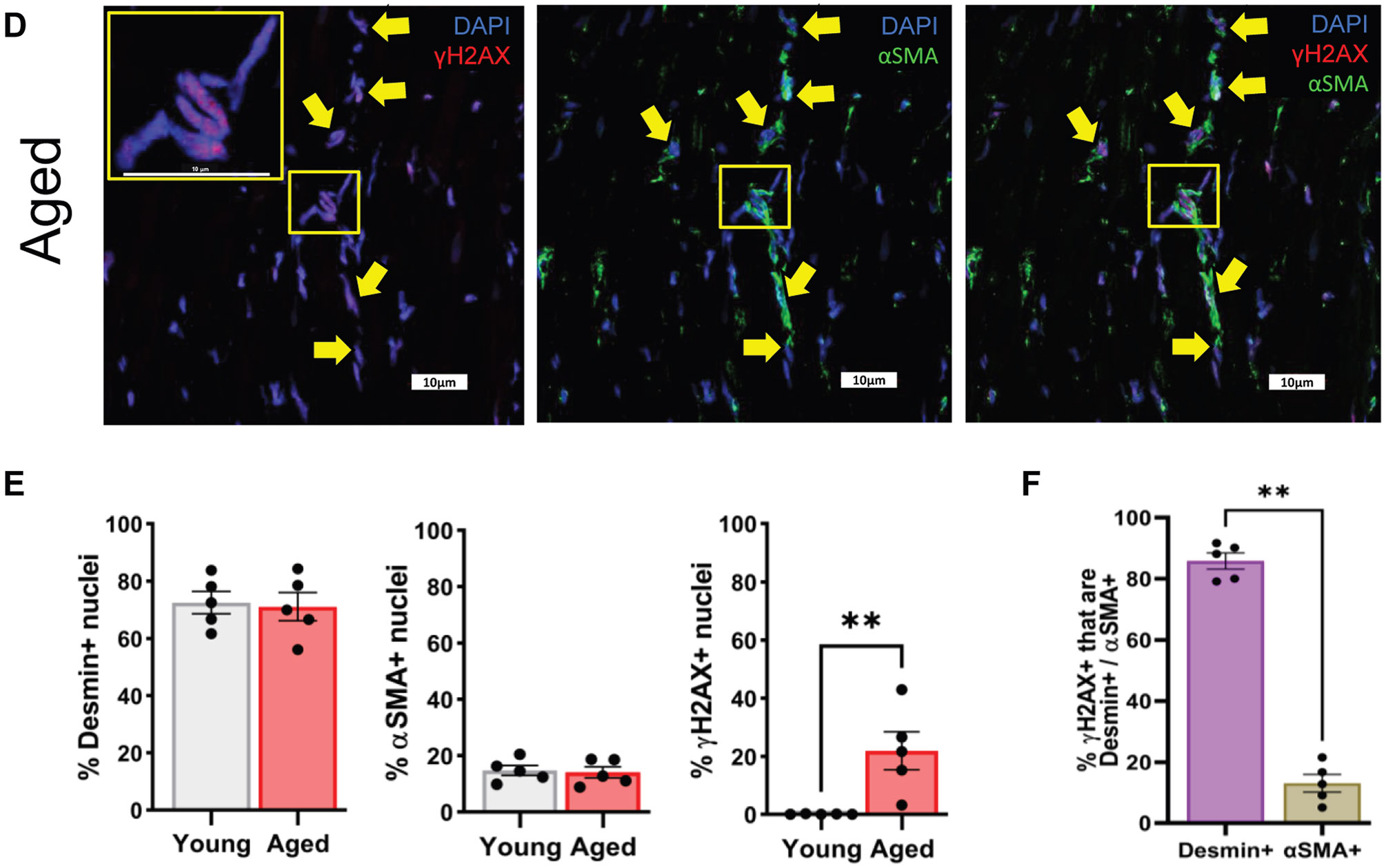
Senescence in the atria of aged and young rabbits. **A:** Representative SA-β-Gal-stained images of aged (4y6m) and young animals (11m). The scale bar of the aged animal’s 2× image is 6 mm. The scale bar of the young animal’s 2× image is 4 mm. Scale bars of 40× images are 60 μm. *Blue color* represents SA-β-Gal positivity. Frozen sections were fixed in 0.5% glutaraldehyde, stained with X-gal at 37 ° C (no CO_2_) for 16 hours, and counterstained with nuclear fast red (NFR). **B:** Scatter plot (mean ± SEM) shows the percentage of SA-β-Gal+ cells in aged and young atria. 5 randomly selected representative images at 40× per animal were used for quantification. **P < .01, Mann-Whitney U test (n = 6 aged and n = 7 young rabbits). **C:** Representative IF confocal images showing staining for γH2AX and desmin. *Top:* Images from an aged atrium. *Bottom:* Images from a young atrium (note: no γH2AX+ nuclei were detected in the region depicted from the young atrium). Confocal images (60×, 10 Z-stacks) were acquired using a Nikon Ti2 microscope. Maximum intensity projections were analyzed in ImageJ, considering signal location and morphology. γH2AX positivity was defined as ≥3 nuclear foci overlapping DAPI; non-nuclear or diffuse signals were excluded. **D:** Representative IF confocal images show staining for γH2AX and αSMA in an aged atrium. **C and D:** *Left:* DAPI in *blue* and γH2AX in *red*. *Center:* DAPI in *blue* and desmin in *white* or αSMA in *green*. *Right:* All channels together. All scale bars of confocal images are 10 μm. The *large yellow rectangles* are magnified regions indicated by the smaller yellow rectangles. *Yellow arrows* indicate cells that are positive for both γH2AX and the cell type–specific marker. **E:** Quantification of the total percentage of desmin+, αSMA+, and γH2AX+ nuclei in aged and young atria. **F:** Quantification of the percentage of γH2AX+ nuclei that are either desmin+ or αSMA+ in aged animals. A combined analysis was performed for both atria. n = 5 for aged, n = 5 for young animals. Scatter plots = mean ± SEM, *P < .05, **P < .01, Mann-Whitney U test. The percentage of SA-β-Gal-positive cells was calculated by manually counting SA-β-Gal-positive and SA-β-Gal-negative cells using the counter tool in ImageJ. αSMA = alpha smooth muscle actin; CO_2_ = carbon dioxide; DAPI = 4’,6-diamidino-2-phenylindole; IF = immunofluorescence; LA = left atrium; RA = right atrium; SA-β-Gal = senescence-associated β-galactosidase; SEM = standard error of the mean.

Senescence-associated β-galactosidase (SA-β-Gal) staining was used to determine the fraction of senescent cells in 7 young and 6 aged atria (Figure 4A). The results showed that the LAs and RAs of aged rabbits contain significantly more SA-β-Gal+ cells (aged 17.2% ± 10.3% [RA] and 12.5% ± 9.6% [LA]; young 0.6% ± 1.0% [RA] and 0.2% ± 0.3% [LA]; *P* < .01) (Figure 4B). No significant difference was detected between the LAs and RAs within the same group. However, greater variability was observed within the aged group (SD for RA 10.3% [aged] vs 1.0% [young]; SD for LA 9.6% [aged] vs 0.3% [young]), as reflected in the electrophysiological parameters from the OM data (Figure 2I-2L).

To identify types of senescent cells in the atria, frozen atrial tissue sections were stained for γH2AX, a marker of nuclear DNA damage. Representative images of an aged (top panel) and a young atrium (bottom panel) stained for the myocyte marker desmin and senescence marker γH2AX are presented in Figure 4C. The results show that most of the γH2AX+ nuclei stained positive for myocyte marker desmin. Staining of an aged rabbit atrium stained for the myofibroblast marker alpha smooth muscle actin (αSMA) and the senescence marker γH2AX is presented in Figure 4D. Notably, we counted the number of nuclei, focusing primarily on regions with the highest tissue integrity, and avoided vessel areas. Quantification of the immunofluorescence (IF) results revealed that the young atria had 72.5% ± 8.8% desmin+ and 14.07% ± 4.04% αSMA+ nuclei, whereas aged atria had 71.1% ± 10.9% desmin+ and 14.03% ± 4.5% αSMA+ nuclei (Figure 4E). Importantly, we observed a significantly higher number of γH2AX+ nuclei in aged atria (21.98% ± 14.7% [aged] vs 0.08% ± 0.11% [young]) (*P* < .01). Furthermore, these results demonstrate that 85.8% ± 5.86% of the γH2AX+ nuclei in aged atria belong to myocytes as defined by desmin costaining and their rod shape typical of atrial myocytes (Figure 4F). In addition, 13.04% ± 6.4% of the γH2AX+ nuclei stained positive for αSMA.

### Increased senescence marker expression and inflammatory pathway activation in aged rabbit atria

**Figure 5:**
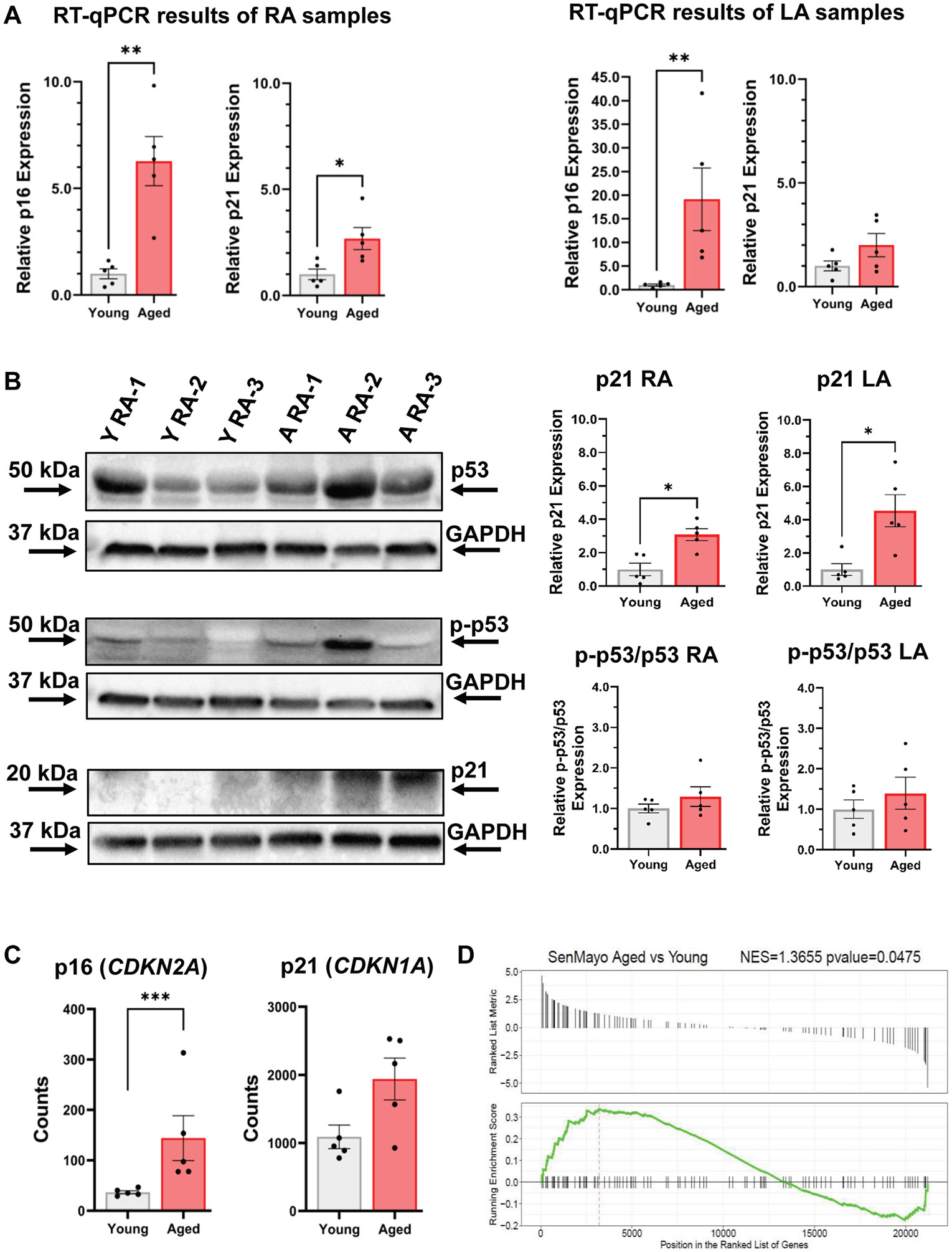

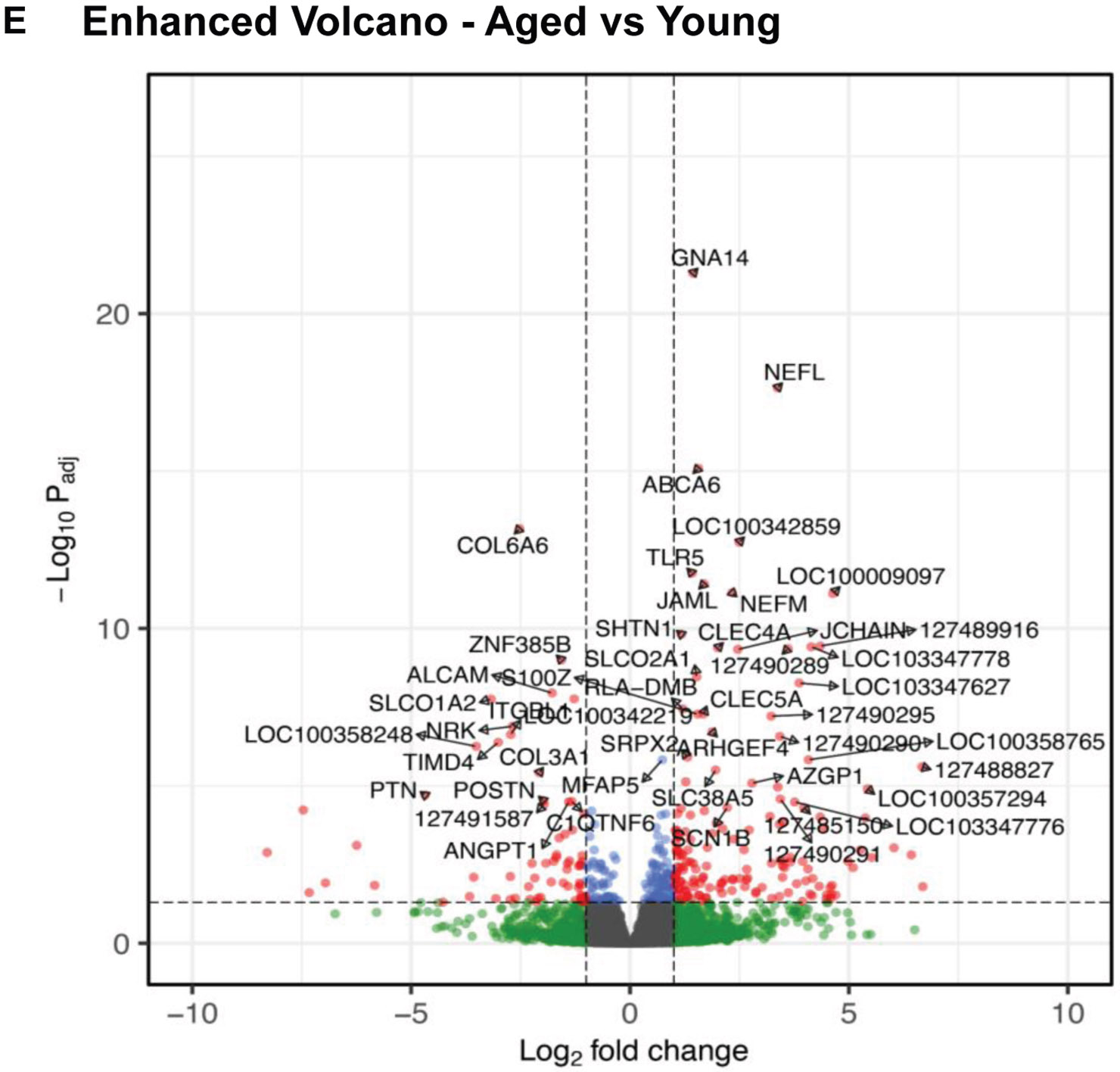
Analysis of gene expression and protein levels. **A:** Scatter plots (mean ± SEM) show relative RNA expression levels of senescence genes p16 (*CDKN2A*) and p21(*CDKN1A*) in the right and left atria of aged and young rabbits. Total RNA from rabbit atrial tissue was extracted with TRIzol, DNAse treated, and quality checked via Bioanalyzer. RT-qPCR was performed with SYBR green using Primer-BLAST-designed primers. SRP14 served as an internal control; expression was analyzed by the ΔCT method. **B:** *Left:* Representative immunoblots of right atria (3 young and 3 aged rabbits) show levels of p53, phospho-p53 (Ser15), p21, and their respective GAPDH. *Right:* Scatter plots (mean ± SEM) show relative protein expression levels of p21, as well as p53 activation (phospho-p53-to-total p53 ratio) in the right and left atria of aged and young rabbits *P < .05, **P < .01, Mann-Whitney U test (n = 5 aged and n = 5 young rabbits). Proteins were extracted from 20–30 mg of rabbit atrial tissue using RIPA buffer with inhibitors. Heat-denatured lysates (40–80 μg, 60°C, 15 minutes) were analyzed by SDS-PAGE. Bands were quantified using ImageLab. **C:** Scatter plots depict RNA level expression differences of senescence genes p16 (*CDKN2A*) and p21(*CDKN1A*) in aged vs young atria (***P < .001). High-quality RNA was isolated using TRIzol and sent to Azenta Life Sciences US, Inc, for library creation and sequencing. Analysis of the raw data was performed inhouse. **D:** GSEA shows that the SenMayo gene set is significantly enriched in aged vs young rabbit right atria. **E:** Volcano plot showing the genes that are significantly upregulated (the right half) and downregulated (the left half) in aged compared with young atria. A = aged; GAPDH = glyceraldehyde-3-phosphate dehydrogenase; GSEA = gene set enrichment analysis; LA = left atrium; NES = normalized enrichment score; RA = right atrium; RIPA = radioimmunoprecipitation assay; RT-qPCR = reverse transcription quantitative polymerase chain reaction; SDS-PAGE = sodium dodecyl sulfate–polyacrylamide gel electrophoresis; SEM = standard error of the mean; Y = young.

We conducted reverse transcription quantitative polymerase chain reaction (qPCR), Western blot, and RNA sequencing (RNAseq) analyses to assess senescence marker expression and inflammatory pathway activation in young and aged rabbit atria. Reverse transcription qPCR revealed that transcript levels of p16 (*CDKN2A*) were 19.1-fold higher (*P* < .01) in aged LAs and 6.3-fold higher (*P* < .01) in aged RAs than young atria. p21 (*CDKN1A*) transcript levels were 2.7-fold higher (*P* < .05) in aged RA, with a similar trend observed in aged LA (Figure 5A). Western blot analysis confirmed that p21 protein levels were elevated 4.5-fold in aged LA (*P* < .05) and 3.08-fold in aged RA (*P* < .05), whereas total p53 and phospho-p53 (serine 15) levels remained unchanged (Figure 5B). A suitable antibody for the rabbit p16 protein was not available.

Bulk RNAseq of RA tissue from aged (n = 5) and young rabbits (n = 5) identified 247 upregulated and 116 downregulated genes in aged animals (false discovery rate < 0.05). Consistent with qPCR results, p16 (*CDKN2A*) expression was significantly higher in aged atria (144.04 ± 99.7 vs 36.7 ± 6.8), whereas p21 (*CDKN1A*) showed a nonsignificant upward trend (*P* = .127) (Figure 5C). The SenMayo gene set,^12^ comprising 125 senescence-related genes, was significantly upregulated (*P* < .05) in aged RA tissue (Figure 5D). Concordantly, gene set enrichment analysis using Kyoto Encyclopedia of Genes and Genomes^13^ and Gene Ontology^14^ pathways revealed significant enrichment of inflammatory response pathways, including both innate and adaptive immune activation. Representative differentially expressed genes are illustrated in the volcano plot (Figure 5E).

### Fibrosis assessments in aged and young rabbit atria

We checked the degree of fibrosis of aged (n = 5) and young atria (n = 7) using Masson’s trichrome (Supplemental Figure 1A). High individual variations within the aged group were observed during the analysis. The results (Supplemental Figure 1B) show that 16.6% ± 12.1% of the aged LAs and 6.1% ± 4.6% of the aged RAs are fibrotic areas, whereas 8.4% ± 2.9% of the young LAs and 9.9% ± 2.6% of the young RAs are fibrotic areas. The differences between the groups did not reach statistical significance.

### Islands of senescent cells in human atrial samples

**Figure 6:**
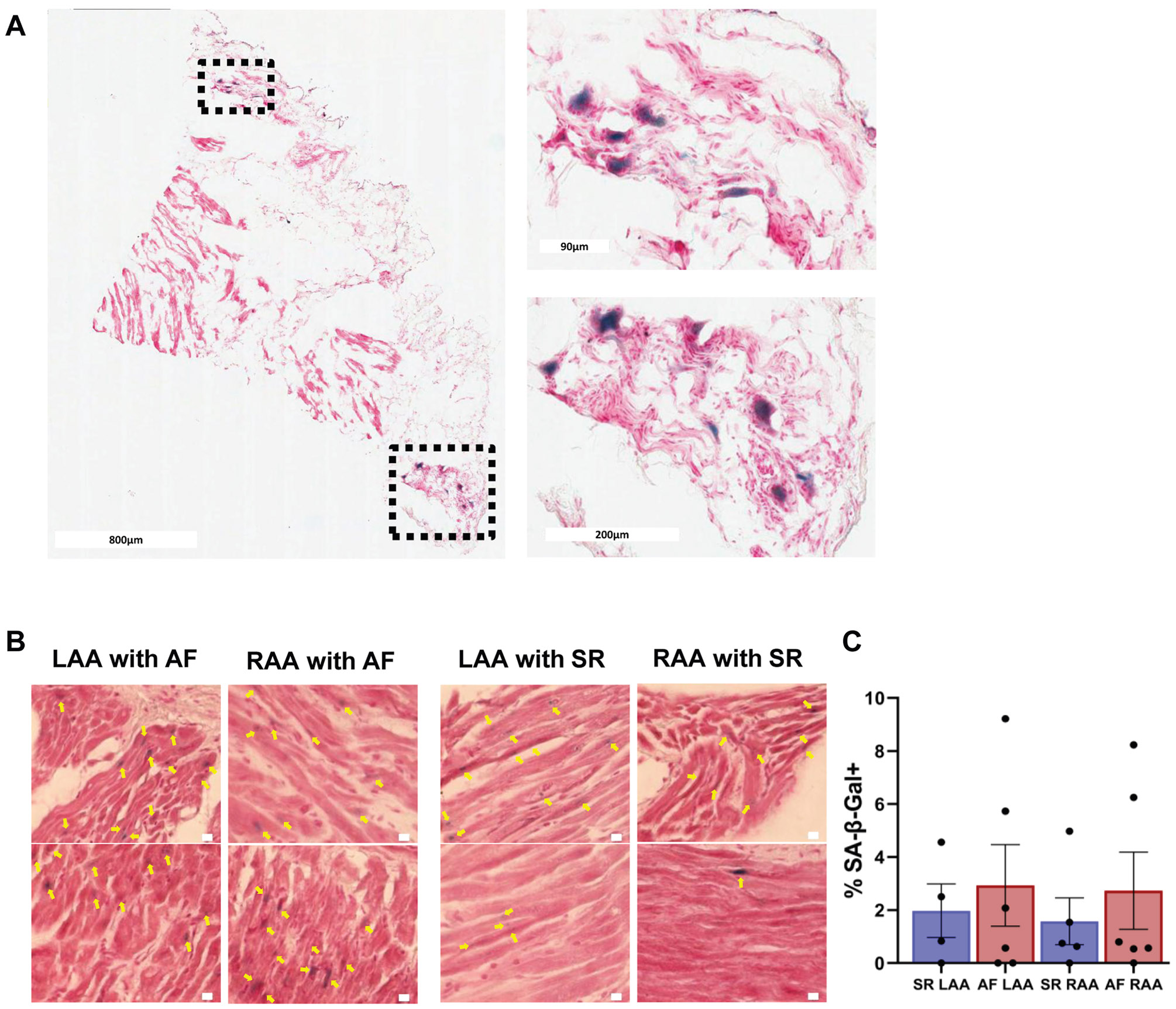
Senescence assessment of human atrial samples with and without AF. **A:** Human left atrial sample from a 54-year-old female patient with SR (sinus rhythm) stained for SA-β-Gal (counterstained with NFR). Magnified senescent cell islands are shown on the right. **B:** Representative SA-β-Gal-stained images of human RAA and LAA samples either with AF or sinus rhythm (counterstained with NFR). *Yellow arrows* indicate areas with SA-β-Gal positivity. White scale bars are 10 μm. **C:** Scatter plot (mean ± SEM) shows the percentage of SA-β-Gal+ cells in the right and left appendages of patients with either SR or AF at the time of surgery. The average age of AF LAA (n = 6) and AF RAA (n = 6) is 63.8 years. The average age of SR LAA (n = 4) is 65.5 years, and SR RAA (n = 5) is 63.6 years. A summary of available patient characteristics, including sex, rhythm status at the time of surgery, and available clinical metadata, is provided in Supplemental Table 1. ANOVA (Kruskal-Wallis test) with Dunn’s post hoc test. AF = atrial fibrillation; ANOVA = analysis of variance; LAA = left atrial appendage; NFR = nuclear fast red; RAA = right atrial appendage; SA-β-Gal = senescence-associated β-galactosidase; SEM = standard error of the mean; SR = sinus rhythm.

We also examined the senescence profile of human atrial tissues. Initial histologic analysis revealed discrete clusters of senescent cells in LA samples from individuals in sinus rhythm (SR) (Figure 6A). We then quantified senescent cell abundance in both left (LAAs) and RA appendages (RAAs) from individuals with and without AF. Not unexpectedly, there was a significant variability in the percentage of senescent cells in these samples: 2.9% ± 3.8% in LAAs derived from individuals with AF (n = 6), 1.98% ± 2.01% in LAAs of individuals with SR (n = 4), 2.7% ± 3.6% in RAAs of individuals with AF (n = 6), and 1.6% ± 1.97% in the RAAs of individuals with SR (n = 5). Overall, there were no statistically significant differences in the percentage of senescent cells between AF and SR groups in either atrial region (Figure 6B and 6C).

### Increased senescent myocytes and fibroblasts in human AF samples

**Figure 7:**
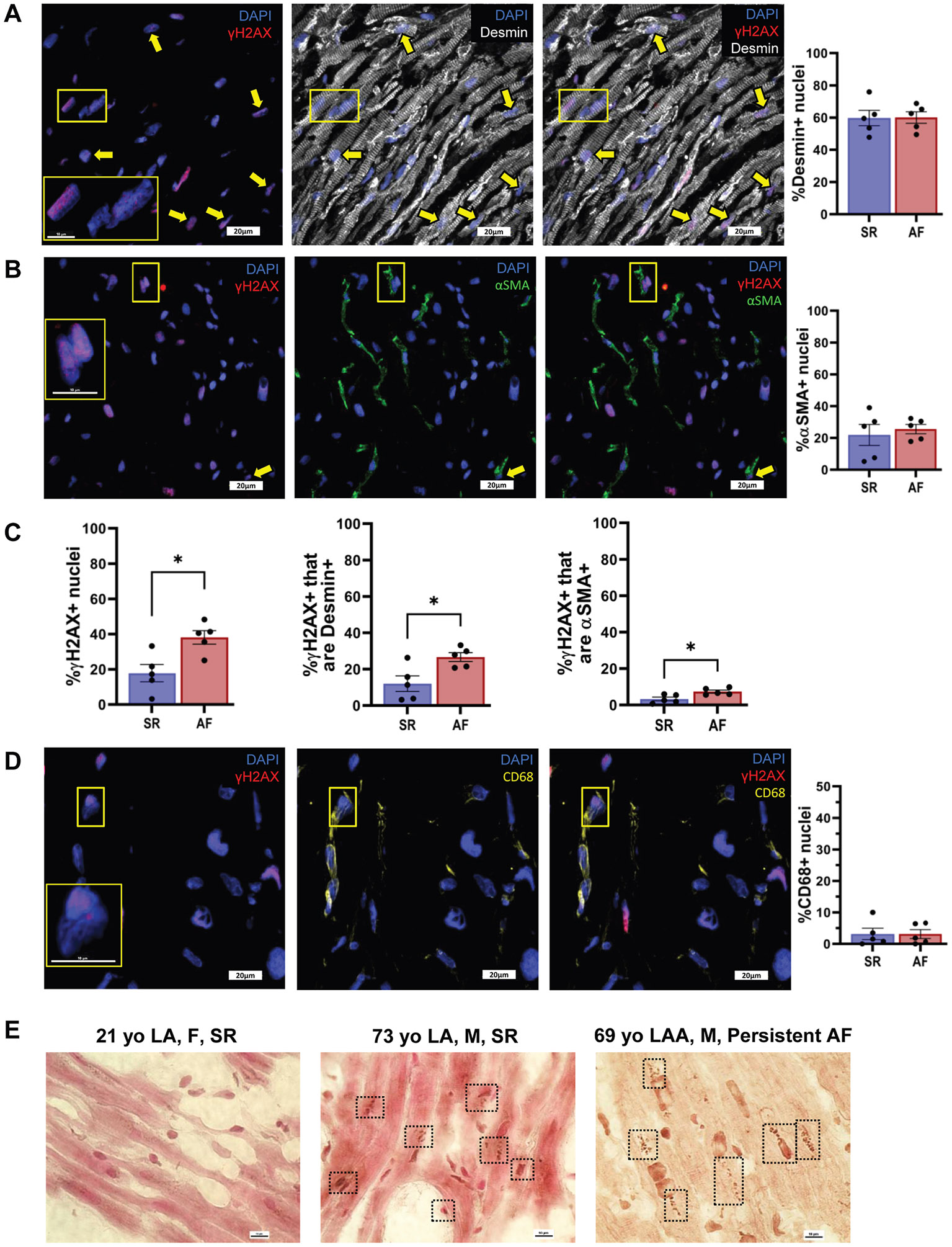
IF and IHC data from human right atrial specimens with and without AF. **A:** Representative IF confocal images at 60× of atria with AF showing staining for γH2AX and desmin. *Right:* Quantification of percent desmin+ nuclei. **B:** Representative IF confocal images of atria with AF showing staining for γH2AX and αSMA. *Right:* Quantification of percent αSMA+ cells. **C:** Quantification of total % γH2AX+ nuclei and % γH2AX+ nuclei that are either desmin+ or αSMA+. **D:** Representative IF confocal images of atria with AF showing staining for γH2AX and CD68. **A, B, and D:** *Left:* DAPI in *blue* and γH2AX in *red*. *Center:* DAPI in *blue* and desmin in *white*, αSMA in *green*, or CD68 in *yellow*. *Right:* All channels together. *Bigger yellow rectangles* show focused areas outlined by the smaller rectangles. *Yellow arrows* indicate cells that are positive for both γH2AX and the cell type–specific marker. Scale bars of confocal desmin and αSMA images are 20 μm. Scale bars of focused nuclear images and all CD68 images are 10 μm. n = 5 for AF, n = 5 for SR. The average age of AF RAA is 65.8 years. The average age of SR RAA is 68.3 years. Scatter plot = mean ± SEM, *P < .05, Mann-Whitney U test. **E:** Representative images of female (F) and male (M) human atrial samples stained for p16. *Black rectangles* indicate the positive areas for the staining. Scale bars are 10 μm. Atrial tissue sections were fixed, blocked, and stained using the VECTASTAIN Elite ABC Kit. Slides were incubated with p16 primary and secondary antibodies (30 minutes each, RT), then counterstained with NFR. AF = atrial fibrillation; αSMA = alpha smooth muscle actin; DAPI = 4′,6-diamidino-2-phenylindole; F = female; IF = immunofluorescence; IHC = immunohistochemistry; LA = left atrium; LAA = left atrial appendage; M = male; NFR = nuclear fast red; RAA = right atrial appendage; RT = room temperature; SEM = standard error of the mean; SR = sinus rhythm.

Anonymized RAA samples from individuals with AF (n = 5) or SR rhythm (n = 5) were analyzed using IF. Representative confocal images of human RA tissue stained for the myocyte marker desmin and the senescence marker γH2AX are shown in Figure 7A. The proportion of desmin-positive nuclei was similar between AF and SR samples (60.1% ± 7.9 vs 59.7% ± 10.6, respectively). Likewise, staining for the myofibroblast marker αSMA (Figure 7B) revealed no significant difference in αSMA+ nuclei between groups (AF 25.6% ± 6.6%; SR 21.9% ± 14.8%).

In contrast, AF samples displayed a significantly higher abundance of γH2AX+ nuclei than SR (38.1% ± 8.6% vs 17.8% ± 10.98%; *P* < .05). This increase was also observed in γH2AX+ nuclei colabeled with desmin (26.6% ± 5.4% [AF] vs 12.04% ± 9.5% [SR]; *P* < .05) and αSMA (7.4% ± 1.9% [AF] vs 3.3% ± 2.3% [SR]; *P* < .05), as shown in Figure 7C. The proportion of CD68+ macrophages/monocytes was low and comparable between groups (AF 3.1% ± 3.2%; SR 3.2% ± 4.03%; *P* = not significant) (Figure 7D). In addition, immunohistochemistry staining for p16 revealed qualitatively increased p16+ cells in aged vs young atrial tissue (Figure 7E); however, owing to the limited number of young human samples (n = 2), no statistical comparison was performed.

### LA diameter correlates with the expression of SASP factors in humans

Bulk RNAseq was performed on LAA tissues from 265 patients who underwent cardiac surgery at the Cleveland Clinic.^15^ The cohort included 181 males and 84 females, with a mean age of 60 ± 12 years; 235 patients were of European and 30 of African descent. To investigate the clinical relevance of senescence, we assessed the correlation between the expression of senescence-associated genes and LA diameter, a well-established risk factor for AF—measured by echocardiography. Several senescence and SASP-related transcripts showed positive correlations with LA diameter in a sex-specific manner. These included matrix metallopeptidase 1, transforming growth factor (TGF)-β2, and endothelin 3 in males; TGF-β1 and tissue inhibitor of metalloproteinase 1 in females; and vascular cell adhesion molecule 1, SERPINE2, and thrombospondin 2 in both sexes (Table 1).

### Fibrosis assessments in human RAAs and LAAs, AF vs SR

We investigated the degree of fibrosis of human samples, which showed that individuals from both groups had a relatively large area of fibrosis in RAA (n = 5) compared with LAA samples (n = 5) (Supplemental Figure 2A and 2B). In particular, the AF group’s relative area of fibrosis of RAA samples was 19.0% ± 7.1% compared with the SR group with 15.2% ± 10.2% fibrosis. The LAA samples showed a relative fibrotic area of 7.7% ± 3.8% (AF) and 7.2% ± 5.9% (SR). The differences between the groups did not reach statistical significance.

### Senolytic treatment reduces atrial senescence and arrhythmia susceptibility in aged rabbits

To evaluate whether pharmacologic clearance of senescent cells could mitigate age-associated atrial remodeling and arrhythmia susceptibility, we performed pilot studies using the naturally occurring senolytic compound fisetin. Fisetin is currently under investigation in multiple human clinical trials and has an established safety profile.^16,17^

**Figure 8:**
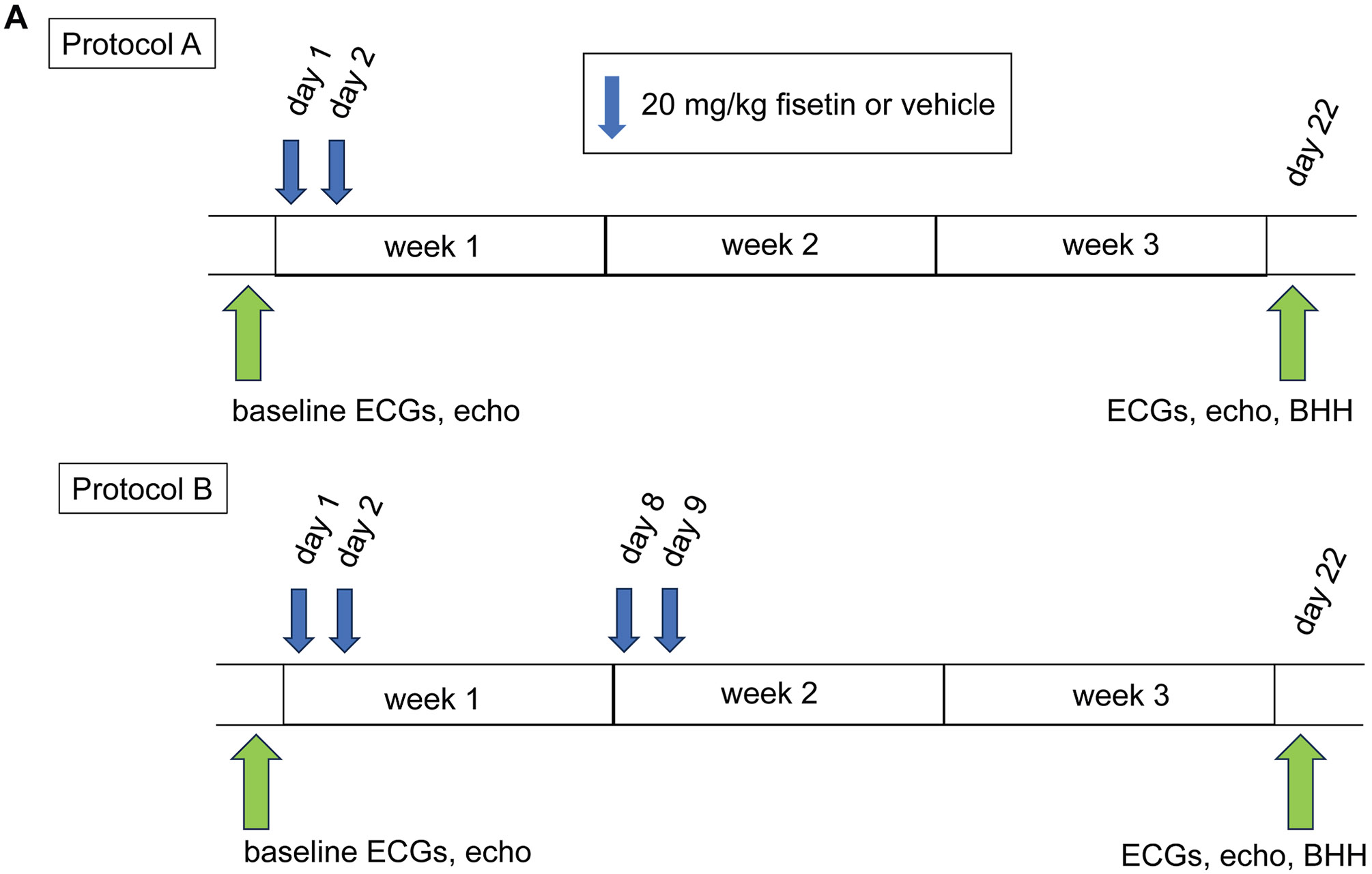

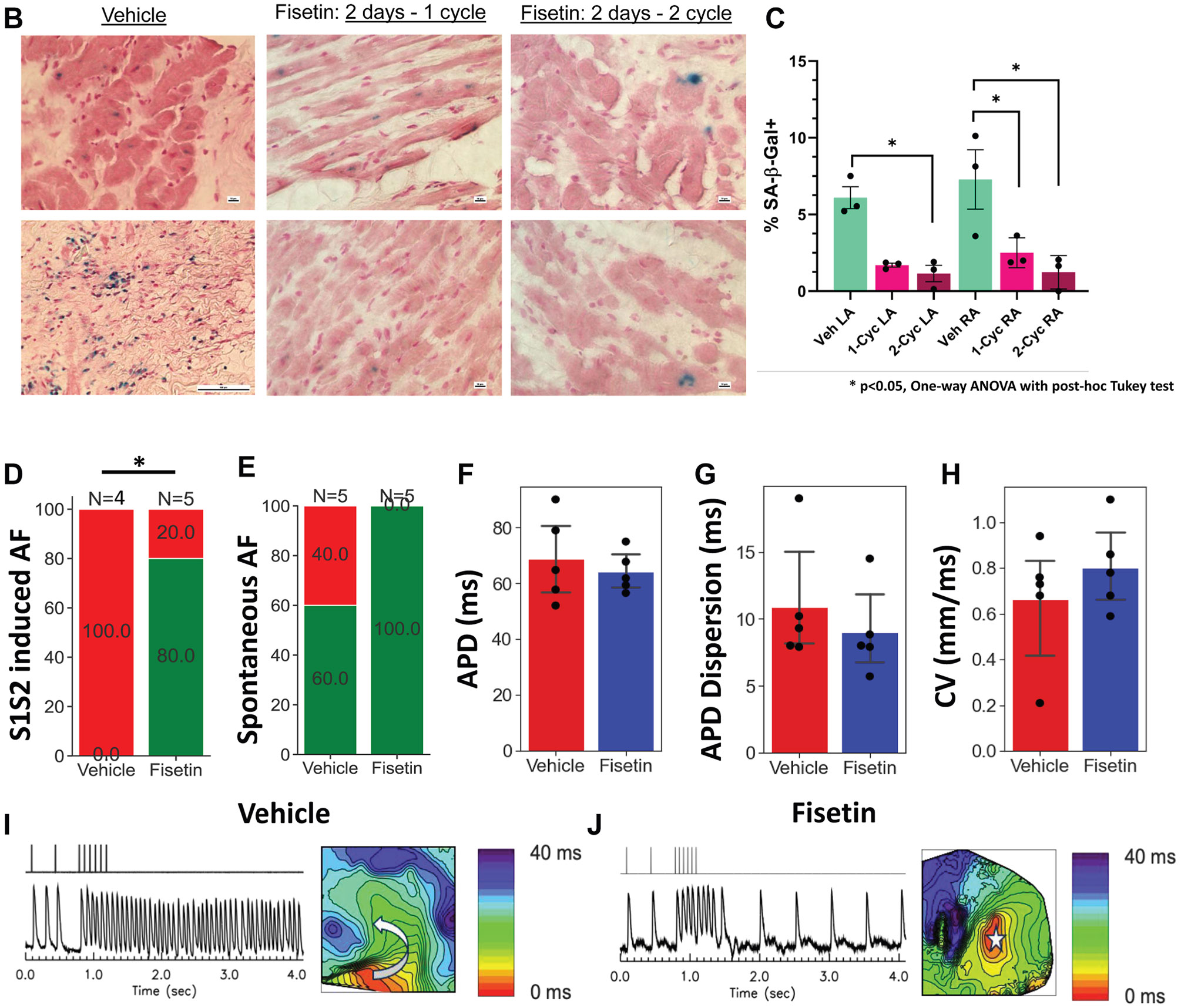
Impact of senolytic treatment on atrial senescence and arrhythmic remodeling in aged rabbits. **A:** Fisetin application protocols. Protocol A: animals received vehicle or fisetin (20 mg/kg) for 2 days throughout the protocol. Protocol B: animals received either fisetin (20 mg/kg) or vehicle 2 days per week and for 2 weekly cycles throughout the protocol. **B:** Senescence assessment of the fisetin treatments of aged rabbits. Representative SA-β-Gal-stained images (counterstained with NFR staining). *Blue color* indicates SA-β-Gal-positive cells. Small scale bars are 10 μm. Big scale bar is 100 μm. **C:** Quantification results of percent SA-β-Gal+ cells. n = 3 for all groups. The average age per group is 4.4 years. Scatter plot: mean ± SEM, *P < .05. 1-way ANOVA with post hoc Tukey test. **D and E:** In the fisetin-treated group, spontaneous AF was absent and S1S2-induced AF was markedly reduced (*red*, AF; *green*, no AF; Fisher’s exact test *P < .05). **F–H:** Characterization of atrial action potential and AF in hearts of fisetin-treated aged rabbits. Preliminary results of APD and CV of fisetin (*blue*) or vehicle hearts (*red*) (n = 5 each). Column plots show mean ± standard deviation. Propagation maps indicate that multiple reentry maintains long-lasting AF in the vehicle group (*white arrow*) (I), whereas several premature atrial contractions (PACs) did not cause reentry and AF were not triggered in the fisetin-treated group (J). AF = atrial fibrillation; ANOVA = analysis of variance; APD = action potential duration; BHH = beating heart harvest; CV = conduction velocity; ECG = electrocardiogram; LA = left atrium; NFR = nuclear fast red; RA = right atrium; SA-β-Gal = senescence-associated β-galactosidase; SEM = standard error of the mean; Veh = vehicle; 1-Cyc = 2 days to 1 week of treatment; 2-Cyc = 2 days to 2 weeks of treatment.

We designed 2 short-term treatment protocols with aged rabbits. In protocol A, 2 consecutive daily doses were followed by a 3-week observation period; in protocol B, 2 weekly cycles of 2 daily doses were followed by 1 week of observation (Figure 8A). ECGs and echocardiograms were obtained before and after treatment. On day 22, atria were harvested and analyzed by SA-β-Gal staining. The results (Figure 8B and 8C) show that fisetin treatment for 2 cycles eliminated most of the senescent cells from the LAs and RAs (from 6.09% ± 1.24% to 1.15% ± 0.9% in the LA and from 7.28% ± 3.34% to 1.23% ± 1.08% in the RA; *P* < .05). By contrast, 1 cycle of 2-day treatment with fisetin failed to significantly eliminate the senescent cells in the LA.

We next applied protocol B to a larger cohort of aged rabbits (n = 11 per group) blindly treated with fisetin or vehicle for 3 weeks. Of note, none of the rabbits had any previously documented AF. Across the full cohort, ECG monitoring detected spontaneous AF in 2 of 11 vehicle-treated rabbits but in none of the 11 fisetin-treated rabbits (Fisher’s exact test, *P* = .476). These 2 rabbits were subjected to OM. 4 fisetin-treated rabbits exhibited premature atrial contractions, but none progressed to AF. ECG and echocardiographic measures of atrial structure and function, including PR, QRS, QT, and R-R intervals, LA strain, and ejection fraction, were not significantly different between groups (data not shown), indicating that fisetin did not produce adverse electrical or structural effects.

A subset of 5 rabbits per group was then evaluated by blinded optical voltage mapping, including the 2 vehicle hearts that displayed spontaneous AF on ECG. Within this OM subset, programmed electrical stimulation induced AF in 4 of 4 vehicle-treated hearts but in only 1 of 5 fisetin-treated hearts (Fisher’s exact test, *P* = .048) (Figure 8D). In addition, 2 vehicle hearts exhibited spontaneous AF during OM, and 1 of these displayed sustained AF throughout the mapping study (Fisher’s exact test, *P* = .444) (Figure 8E). Fisetin treatment did not significantly alter APD, APD dispersion, or CV (Figure 8F-8H), but mapping during programmed electrical stimulation demonstrated fewer reentry events and reduced AF maintenance in fisetin-treated hearts (Figure 8I and J).

## Discussion

In the present study, we detected prolonged APD in aged atrial myocytes and documented both spontaneous and inducible AF in aged rabbits, confirming that this model replicates key clinical characteristics of AF in elderly humans. We further investigated whether aging promotes the accumulation of senescent atrial myocytes and myofibroblasts, creating a proarrhythmic, inflammatory microenvironment. We found a markedly increased senescent cell burden in both rabbit and human atria, accompanied by upregulation of inflammatory and SASP-related gene programs in aged rabbit atria. Importantly, selective clearance of senescent cells with the senolytic compound fisetin markedly reduced senescent cell abundance and arrhythmia susceptibility, supporting a causal contribution of senescence to age-related atrial remodeling.

In humans, the prevalence of AF increases markedly with age. For example, 1 cohort of patients aged ≥65 years showed a prevalence of 22.4% at age 80–84 and 28.5% at age ≥85 years.^18^ Thus, our rabbit model prevalence of ~12.5% falls within the lower range of aged-human values (Figure 1). This finding is significant, given that naturally occurring spontaneous AF in mammals is rare and was only reported for dogs and horses but not for pigs.^19^ In isolated rabbit hearts, spontaneous AF episodes lasted several minutes on average, with 1 episode exceeding an hour. These findings further support the clinical relevance of the model for sustained AF. We also noted a heterogeneous distribution of senescent cells throughout the aged rabbit atria (Figure 4), likely owing to the presence of senescent “zombie cells” that induce senescence in neighboring cells in a paracrine manner.^20^ Importantly, our OM data suggest that tissue heterogeneity may be the primary mechanism contributing to arrhythmia risk in the aging rabbit, as shown with increased APD dispersion, heterogeneous conduction maps, and S1S2-induced reentry formation (Figure 2). These data also underscore previous findings^21^ that suggested that APD heterogeneity in transgenic mice and humans was 1 mechanism by which AF could be initiated. Our OM data are in line with the heterogeneous and accumulated distribution of senescence cells in the aged atria (Figure 4). Tissue heterogeneity along with biological variation in aged atria causes difficulties in obtaining statistical significance in macroparameters such as APD and CV given that heterogeneity and large variation can mask local abnormalities to promote conduction block and reentry formation. Future studies should seek to directly link the locations of focal activity or conduction blocks to the population of senescent cells. As the prevalence of AF increases dramatically with aging, the aged rabbit model is an appropriate animal model to investigate the pathogenesis of age-related spontaneous/inducible AF.

Our patch-clamp studies determined that, relative to young rabbit atrial myocytes, aged rabbit atrial myocytes showed significantly prolonged APD at 30% repolarization level, APD at 50% repolarization level, and APD_90_ (Figure 3). These changes support the presence of a proarrhythmic substrate for arrhythmia in the aged atrium. Shortened APD is more commonly associated with AF, but a recent study in the LAs of aged rabbits showed prolonged APD_90_,^22^ as well as an older study in aged dogs with prolonged APD_90_ in RA tissue compared with young.^23^ However, neither study analyzed the atrial tissue for cellular senescence. In addition, senescent ventricular cardiomyocytes produced from human induced pluripotent stem cells replicated the phenotype of aged cardiomyocytes in terms of senescence markers, electrical and Ca^2+^ handling characteristics (eg, prolonged multicellular corrected QT interval and single-cell APD, with prolonged APD variability and increased frequency of delayed-afterdepolarization incidence), and metabolic features.^24^

Histologic and molecular analyses revealed a robust increase in senescent cell burden in aged rabbit atria. Similar to aged rabbits (Figure 4A), senescent cell accumulation in human atrial samples was heterogeneous, with some areas lacking SA-β-Gal+ signals (Figure 6A and 6B). Quantitative analysis revealed a 10–20-fold increase in senescent cells in aged rabbit atria (Figure 4B). In human AF samples, SA-β-Gal+ cell numbers showed a trend toward an increase but varied significantly (1%–9%), with no statistical difference between AF and SR groups (Figure 6C). As in aged rabbit atria (Figure 4E), human AF samples showed significantly greater γH2AX+ signals than SR samples (Figure 7C). Interestingly, γH2AX+ nuclei were approximately 10-fold more abundant than SA-β-Gal+ cells in human samples (cf. Figures 6C and 7C). This discrepancy may stem from the loss of β-galactosidase activity during tissue processing or indicate that γH2AX+ cells with low DNA damage levels have not yet become senescent.

In both species, cardiomyocytes showed higher senescence levels than fibroblasts. It is important to note that quantification of our IF data was done by counting the nuclei that are positive for the markers. Cardiomyocytes constitute ~70%–80% of cellular volume and ~30%–40% of total heart cells, whereas approximately 90% of rabbit and 25% of human cardiomyocytes are multinucleated.^25^ The mechanisms by which senescence pathways are initiated and governed in multinucleated cells, such as cardiomyocytes, justify further research.

Previous studies suggested that replicative senescence in proliferative cells depends on telomere shortening, but more recent evidence shows that postmitotic cells can also undergo stress-induced premature senescence triggered by DNA damage and oxidative stress, independent of telomere length.^26,27^ Oxidative and mitochondrial stress are known contributors to AF progression,^28,29^ and persistent DNA damage can induce senescence even in cells with long telomeres.^30-32^ A recent study demonstrated that telomere-associated DNA damage in purified cardiomyocytes promotes senescence with a distinct SASP profile, including factors such as growth differentiation factor 15, endothelin 3, and TGF-β2, implicating senescent cells in cardiac remodeling beyond inflammation.^33^

We also confirmed the presence of senescent endothelial cells (data not shown) and macrophages in human atrial tissue (Figure 7), thus corroborating literature showing different types of senescent cells found in human atria.^34-36^ These findings parallel the senescence patterns seen in aged rabbit atria, reinforcing the translational relevance of our model and supporting the notion that conserved molecular mechanisms underlie atrial aging across species.

In our study, immunohistochemical staining showed increased p16 signal in aged human atrial tissue (AF and SR), suggesting a higher burden of senescent cells with age (Figure 7E). Although statistical power was limited for comparisons owing to sample availability, the observed trends are consistent with the elevated expression of senescence-associated genes (p16, p21) and SASP factors, such as beta-galactosidase 1 and growth differentiation factor 15, in human RAA samples with aging.^37^ Recent genome-wide association studies have identified p21 (*CDKN1A*) as an AF risk gene.^38,39^

At the molecular level, aged atria displayed upregulation of canonical senescence markers p16 and p21, and our RNAseq data showed upregulation of inflammatory pathways and the innate immunity response, as well as genes related to complement and coagulation cascades (Figure 5). These transcriptional changes are consistent with the SASP, characterized by the release of cytokines and proteases that promote tissue inflammation and matrix remodeling.

Atrial fibrosis provides a key substrate for AF maintenance and can be promoted by SASP factors such as tumor necrosis factor α, TGF-β, nuclear factor kappa B, interleukins, and matrix metallopeptidases, which contribute to inflammation and fibroblast activation. Previous studies have reported increased fibrosis and shorter telomeres in atrial tissue from patients with AF, with senescent fibroblasts as the predominant cell type.^40^ However, many samples in those studies were obtained from patients with valvular disease, limiting generalization to age-related AF.^35^ In our study, atrial fibrosis showed a nonsignificant trend toward an increase in aged rabbits and human AF samples (Supplemental Figures 1 and 2), consistent with high interindividual variability. Notably, emerging evidence indicates that the spatial distribution and heterogeneity of fibrosis, rather than total fibrotic burden, may be the key determinant of arrhythmogenic substrate formation.^41^

Atrial dilatation is a well-known risk factor for AF, reflecting structural and electrical remodeling of the atria that are important AF substrates.^42^ Our gender-specific analysis of bulk RNAseq results from human LAA tissues revealed a correlation between LA diameter and upregulation of some well-known SASP factors (Table 1). With senescent cells being a potential source for the secretion of SASP factors, increased expression of these factors could further exacerbate atrial remodeling and fibrosis, creating a vicious cycle that promotes AF progression and maintenance.

To bridge our mechanistic findings with clinical relevance, we asked whether the selective elimination of senescent cells could prevent the development of the arrhythmogenic substrate in aged atria. Importantly, all senescence assessments before pharmacologic intervention were performed in aged rabbits that had not developed spontaneous AF; animals that did exhibit AF were used exclusively for electrophysiological studies and were not included in biochemical or histologic analyses. Short-term treatment effectively removed most senescent cells in the atria without adverse effects on cardiac structure or function (Figure 8). Spontaneous and inducible AF occurred only in vehicle-treated animals, whereas fisetin-treated rabbits remained free of sustained arrhythmias, despite the presence of occasional premature atrial contractions. Our findings demonstrate, for the first time in a large-animal model, that pharmacologic clearance of senescent cells mitigates the arrhythmogenic substrate associated with aging.

OM corroborated these observations: hearts from fisetin-treated rabbits exhibited reduced reentry formation and significantly lower arrhythmia inducibility, consistent with normalization of conduction heterogeneity and decreased spatial dispersion of repolarization. The concordant electrophysiological and histologic outcomes provide compelling evidence that senescence is not a secondary consequence of AF but an upstream, modifiable driver of atrial remodeling. Notably, preliminary extended fisetin treatment, consisting of repeated monthly doses over 5 months, seemed to maintain downregulation of inflammatory and senescence-associated pathways (including tumor necrosis factor α/nuclear factor kappa B signaling and the SenMayo gene set) while upregulating apoptotic programs, indicating prolonged suppression of proinflammatory and senescence signatures (data not shown). These findings support the notion that senescent cells perpetuate atrial inflammation through SASP activity and that their clearance can provide durable anti-inflammatory effects.

Together, our data establish cellular senescence as a mechanistic link among aging, inflammation, and AF and provide functional proof that its targeted elimination can prevent the emergence of an arrhythmogenic substrate. This preventative effect differentiates senolytic therapy from current rhythm- or rate-control strategies, which primarily address downstream electrophysiological manifestations. By acting upstream of structural and inflammatory remodeling, senolytics such as fisetin may offer a mechanism-based approach to maintain atrial integrity and reduce AF incidence in the aging population. The favorable safety profile of fisetin and its ongoing human trials for other age-related conditions further enhance its translational promise.

A deeper understanding of how senescent cells orchestrate inflammatory and electrical remodeling will inform future preventative and disease-modifying therapies for AF. Integrating senolytic or senomorphic interventions with established rhythm-control approaches could represent a paradigm shift—from managing arrhythmia recurrence to preserving atrial health before irreversible remodeling occurs.

### Future directions and limitations of the study

Several nonsignificant findings may reflect inherent biological variability; we acknowledge that certain endpoints may require larger cohorts to definitively exclude subtle effects. Owing to budgetary constraints, the fisetin studies were conducted with small numbers of aging rabbits, which may have limited the statistical power required to detect biologically relevant differences.

In addition, although we observed increased expression of SASP factors in aged atrial tissue, which are known mediators of inflammation and fibrosis, direct assessments of their role in inflammatory signaling are beyond the scope of this study. Future work should incorporate cytokine profiling, immune cell characterization, and temporal resolution of senescence and inflammatory activation. Studies that directly probe paracrine and cell-cell interactions between senescent fibroblasts and cardiomyocytes will be critical to clarify mechanistic links among senescence, atrial remodeling, and arrhythmogenesis.

This study was designed to investigate aging-associated cellular senescence as an upstream biological substrate that increases atrial vulnerability to arrhythmia, rather than atrial remodeling secondary to sustained AF. Accordingly, the analysis of human atrial tissue did not distinguish aging-related from AF-associated senescence, which should be considered when interpreting these findings. Importantly, our data demonstrate that senolytic therapy reduces inducible AF in the aging rabbit heart, supporting a contributory role for senescence in arrhythmia susceptibility. We do recognize that AF itself may further promote cellular senescence, consistent with previous reports,^34^ suggesting a bidirectional relationship that warrants further investigation.

Because there is a paucity of commercially available retired male breeders, all experiments were performed using female rabbits. There are well-documented sex differences in AF (reviewed in Tamirisa et al^43^). In addition, multiple studies suggest that the reproductive burden in women may lead to accelerated aging, as indicated by a decrease in telomere length.^44^ Thus, future studies are warranted to study senescence and AF in aged male rabbits.

## Supplementary Material

Supplemental Material

Supplemental Figure 1

Supplemental Figure 2

## Figures and Tables

**Table 1:** Correlations between left atrial (LA) diameter and canonical SASP factors by gender

SASP factors	F	M	SASP factors	F	M
TP53	.127*	.0013^†^	VCAM1	.0191^†^	.0001^†^
MMP1	.1345*	.0178^†^	SERPINE1	.0579*	.009^†^
MMP9	.5296*	.004^†^	SERPINE2	.0048^†^	<.0001^†^
TGF-β1	.0334^†^	.175*	THBS2	.0006^†^	<.0001^†^
TNF	.7091*	.0817*	FLNA	.0469^†^	.0238^‡^
IL-18	.6232*	.0512*	TFPI	.0085^†^	<.0001^†^
IL-1α	.0803*	.0387^†^	CALU	.0252^†^	.0001^†^
IL-1β	.0765*	.0341^†^	IRF7	.4686*	.0333^‡^
TIMP1	.0253^†^	.1233*	OAS2	.1092*	.0985*
TIMP2	.0653*	.015^†^	AGTR1	.0264^‡^	.4834*
TGF-β2	.8137*	.0014^†^	RB1	.0401^†^	.0302^†^
EDN3	.6068*	.0337^‡^	MT-CO2	.4084*	.0002^‡^

Numbers represent correlation coefficients (*P* values).

AGTR1 = angiotensin II receptor type 1; CALU = calumenin; EDN3 = endothelin 3; F = female; FLNA = filamin A; IL-1α = interleukin-1α; IL-1β = interleukin-1β; IL-18 = interleukin-18; IRF7 = interferon regulatory factor 7; M = male; MMP1 = matrix metallopeptidase 1; MMP9 = matrix metallopeptidase 9; MT-CO2 = mitochondrially encoded cytochrome C oxidase 2; OAS2 = 2’-5’-oligoadenylate synthetase 2; RB1 = retinoblastoma 1; SASP = senescence-associated secretory phenotype; TFPI = tissue factor pathway inhibitor; TGF-β1 = transforming growth factor-beta 1; TGF-β2 = transforming growth factor-β2; THBS2 = thrombospondin 2; TIMP1 = tissue inhibitor of metalloproteinase 1; TIMP2 = = tissue inhibitor of metalloproteinase 2; TNF = tumor necrosis factor; TP53 = tumor protein 53; VCAM1 = vascular cell adhesion molecule 1.

* Nonsignificant *P* values (*P* < .05).

† Positive correlation.

‡ Negative correlation.

## Appendix

### Supplementary data

Supplementary data associated with this article can be found in the online version at https://doi.org/10.1016/j.hrthm.2026.01.007.

## Abbreviations

AF: atrial fibrillation
APD: action potential duration
CV: conduction velocity
LAA: left atrial appendage
OM: optical mapping
RAA: right atrial appendage
SA-β-Gal: senescence-associated β-galactosidase
SASP: senescence-associated secretory phenotype
