# Supplementary material for: Senolytic reduction of senescent cells mitigates atrial arrhythmia vulnerability in aging rabbits
Source: Heart Rhythm. Author manuscript; Available in PMC 2026 Apr 13. (PMC13075518; doi:10.1016/j.hrthm.2026.01.007)
Supplement: Supplemental Material [file NIHMS2158865-supplement-Supplemental_Material.docx]

**Supplemental Material**

**Senolytic Reduction of Senescent Cells Mitigates Atrial Arrhythmia Vulnerability in Aging Rabbits**

**Contents**

Methods

Supplementary Tables 1 – 4

Supplementary Figures S1 – S2

# Methods

**Supplemental Table 1: Human sample characteristics**

| **Characteristic** | **AF Patients (n = 6)** | **SR Patients (n = 5)** |
| --- | --- | --- |
| Age, years | 63.8 ± 8.7 | 63.6 ± 12.2 |
| Sex (Male/Female) | 4 / 2 | 3 / 2 |
| Race (White) | 6 (100%) | 5 (100%) |
| Hypertension | 1 (16.7%) | 0 (0%) |
| BMI, kg/m² | 28.6 ± 4.0 | 27.4 ± 4.2 |
| Diabetes Mellitus | 0 (0%) | 1 (20%)* |
| Smoker | 3 (50%) | 3 (60%) |
| LVEF, % | 58.4 ± 4.3** | 61.3 ± 6.5*** |
| LA Size, cm | 6.4 ± 1.5^†^ | 5.6 ± 0.3^††^ |

* One patient reported diet-controlled diabetes.

** LVEF available from 5 of 6 AF patients.

*** LVEF available from 4 of 5 SR patients.

† LA size available from 4 of 6 AF patients.

†† LA size available from 3 of 5 SR patients

*Tissues*. Young (≤1 year) and aged (≥4 years) female New Zealand white rabbits (*Oryctolagus cuniculus*) were acquired from Robinson Services Inc. (RSI, NC, USA). Rabbits were anesthetized with ketamine (60 mg/kg), xylazine (15 mg/kg IM), buprenorphine (0.03 mg/kg SQ), and sodium pentobarbital (150 mg/kg IV). Hearts were removed as previously described^1^ and washed in cold phosphate buffered saline (PBS). Gross anatomy photos of the whole heart were taken before any dissection. Cardiac tissues for histology experiments were embedded in optimal cutting temperature (OCT) compound solution (Fisher Scientific, Waltham, MA, USA) followed by rapid freezing on liquid nitrogen. Cardiac tissues for biochemical experiments were snap frozen in liquid nitrogen. All samples were stored immediately at -80°C. Cryosectioning was performed at 5 µm thickness for immunofluorescence (IF), 6 µm for immunohistochemistry (IHC), and 10 µm for senescence-associated β-galactosidase (SA-β-Gal) and Masson’s Trichrome staining using a Leica CM1950. Slides were immediately fixed in cold 4% paraformaldehyde (PFA) in PBS for 10 minutes followed by a wash in PBS for 10 minutes for IF, IHC, and Masson’s Trichrome. For SA-β-Gal, slides were fixed in 0.5% glutaraldehyde for 10 minutes followed by a wash in PBS for 10 minutes.

*Electrocardiogram.* Rabbits were anesthetized with a subcutaneous or intramuscular injection of ketamine (25 mg/kg) and xylazine (3.5–3.75 mg/kg). Once adequately sedated, the chest, as well as areas over the left and right shoulders and the left and right hind limbs, were shaved to facilitate electrode placement. Electrodes coated with Hollister medical adhesive were then attached to these sites. A standard 12-lead electrocardiogram (ECG) was recorded, and multiple parameters were measured. ECG recording continued for 10–15 minutes. Following the procedure, rabbits were allowed to recover naturally or were administered atipamezole (0.5 mg/kg, intramuscularly) to accelerate recovery. Spontaneous AF episodes were identified based on irregular R–R intervals, absence of discernible P waves, and rapid atrial activity consistent with AF. To ensure consistency, two independent, blinded observers analyzed the recordings.

*Echocardiogram*. Echo was performed by a PGY-6 cardiology fellow undergoing level 3 echocardiography certification. Rabbits were sedated and excess fur was removed prior to imaging. Echocardiographic measurements: Mitral valve E and A waves, e’ septal and e’ lateral, E/e’ (mean), Left atrial volume (Simpson’s biplane), Left ventricular ejection fraction by Simpson’s biplane, Isovolumic relaxation time, Left atrial global longitudinal strain (reservoir strain), TAPSE, RV S’, Right ventricle global longitudinal strain, Tricuspid regurgitation maximum velocity. The analysis was done using TomTec (TOMTEC Imaging Systems) software.

*Adult Rabbit Cardiac Myocyte Isolation*. The heart was removed from anesthetized young or aged rabbits and perfused for 5 minutes (20 mL/min) with a basic buffer (130mM NaCl, 4.5 mM KCl, 3.5 mM MgCl_2_, 0.4 mM NaH_2_PO_4_, 5 mM HEPES, and 10 mM Glucose), to which 0.75 mM CaCl_2_ was added. After 8 minutes, 0.1 mM EGTA was added to the buffer. Subsequently the heart was perfused with enzyme buffer (basic buffer with 216 or 432 U/mL collagenase I (Worthington Biochemical), 0.52 or 1.08 U/mL Protease XIV, and 240 or 480 µM CaCl_2_). The left and right atria were cut off from the heart, minced into fine pieces, and then transferred into a small beaker that contained 2.0 mL of enzyme buffer and 2% bovine serum albumin (BSA) (Sigma, Cat# A7638-25G). The beaker was then incubated in a 37°C water bath shaker at 137 revolutions per minute. A drop of the supernatant was checked under the microscope every 3-4 minutes until single atrial cells appeared. The supernatant was collected by filtering through a 100 µm cell strainer into a 50 mL tube that contained 1.5 mL of potassium buffer (KB) (85 mM KCl, 5 mM MgSO_4_, 30 mM KH_2_PO_4_, 5 mM Na_2_ATP, 5 mM pyruvate Na, 5 mM DL-ß-hydroxybutyric acid sodium salt, 5 mM phosphocreatine di(tris) salt, 20 mM taurine, 0.56 mM EGTA, 50 g/L PVP 40, 0.1 mM CaCl_2_, and 20 mM glucose), to which 3% BSA was added. The tissue was rinsed off from the strainer into a beaker with 1.5 mL of fresh enzyme buffer and 2% BSA to continue incubation. The collection was repeated every 5 minutes until enough single, rod-shaped myocytes were acquired. All fractions were combined and filtered again into a 50 mL tube. The tube was spun down at 80g for 4 minutes, and the pellet was resuspended in a 15 mL tube with 4-5 mL of KB. Filtration was done again if needed. The 15 mL tube was centrifuged at 80g for two minutes and the pellet was again resuspended in 7-8 mL of KB. Myocytes were let sit in the buffer with a loose cap for 30-45 min to recover before use.

*Optical Mapping Procedure and Data Analysis*. All female young (n=8, 5~7 months old) and aged rabbits (n=13, 4~5 years old) were euthanized with buprenorphine (0.03 mg/kg IM), acepromazine (0.5 mg/kg IM), xylazine (15 mg/g IM), ketamine (60 mg/kg IM), pentothal (35 mg/kg IV), and heparin (200 U/kg). Hearts were excised from the chest and perfused in a Langendorff perfusion apparatus (Radnoti LLC, Covina, CA). Blebbistatin (10 µmol/L) was perfused to reduce movement artifacts^2^. Hearts were stained with the voltage-sensitive dye di-4-ANEPPS, and ECG and perfusion pressure were constantly monitored (PowerLab, AD Instruments, Sydney, Australia). A dual camera system (Ultima-L, Scimedia, Japan) was used to record RA and LA simultaneously. The pacing electrode was placed on the SA nodal region to test vulnerability to AF under stimulation protocol. The hearts underwent two stimulation protocols: 1) S1S1 basic cycle length (CL=350 ms) followed by a single premature S2 beat to reduce S2 cycle length (CL) by 10 ms for APD restitution and tissue refractory period or atrial tachycardia (AT)/AF induction^3^, and (2) S1S1 basic cycle length (CL=350 ms) followed by five premature S2 beats to reduce CL of S2 beat by 10 ms until it reaches refractoriness or AT/AF induction. The data were analyzed with a custom software program developed in Interactive Data Language (Harris Geospatial Solutions). Action potential duration variables and APD maps were calculated via the difference between activation and repolarization time points, determined from fluorescence (F) signals by calculating (dF/dt)max and 75% depolarization (9x9 pixels). Data were filtered using a temporal polynomial filter (3rd order, 13 points). Action potential conduction velocity was calculated using the spatial gradient of activation time (11x11 pixels) and spatial resolution of acquired image (200 µm/pixel).

*Electrophysiology Recording of Action Potentials via Patch Clamping*. Whole-cell patch clamp recordings were obtained from freshly dissociated rabbit myocytes. All experiments were conducted in the whole-cell configuration at 35–37°C with Axopatch-200B, Digidata 1440A, and pClamp 10 software (Molecular Devices). After the cell membrane was broken by suction, the cell membrane capacitance and series resistance were compensated by 70–80% in the voltage clamp-mode. Tyrode solution (in mM): 140 NaCl, 5.4 KCl, 1.8 CaCl2, 1 MgCl2, 0.33 NaH2PO4, 5.5 glucose, and 10 HEPES, pH 7.4 adjusted with NaOH, was used as a standard bath solution. The pipette solution contained (in mM): 100 potassium aspartate, 25 KCl, 10 NaCl, 0.01 EGTA, 10 HEPES, 3 MgATP, 0.002 cAMP-Na, and 10 phosphocreatine di(tris) (pH 7.3 adjusted with KOH). AP was activated in the current clamp mode by a 3 ms injection of a depolarizing current, which was 20% higher than the threshold of AP activation. The AP stimulation rate was 1.25 Hz. The voltage output was filtered by a low-pass filter with a cut-off frequency of 10 kHz and sampled at 20 kHz. AP parameters were calculated using Python 3.11 from the last 10 of 40 stimulated action potentials to ensure steady-state AP morphology. For each atrial myocyte, the 10 trace parameters were averaged, and then plotted with the mean across all cells. Prior to plotting, points for each parameter were removed if they were greater than the upper bound (UB=Quantile1-(1.5*Interquartile range)) or less than the lower bound (LB=Quantile3+(1.5*Interquartile range)). Two-tailed t-test was used for analysis.

*Senescence-associated ß-Galactosidase Assay*. The procedure for SA-β-Gal staining was modified from that previously described^4^. Immediately after cryosectioning, frozen sections of rabbit or human atrial tissues were fixed in 0.5% glutaraldehyde (in 1x PBS) for 10 minutes and then placed in 1x PBS for 10 minutes. Sections were kept at -20°C for same-day staining. For staining, sections were incubated with fresh β-galactosidase staining solution (40 mM citric acid/Na phosphate buffer, 5 mM K_4_[Fe(CN)_6_]·3H_2_O, 5 mM K_3_[Fe(CN)_6_], 150 mM NaCl, 2 mM MgCl_2_, and 1 mg/ml X-gal in dimethylformamide) at 37°C with no CO_2_ for 16 hours. Nuclear Fast Red (Vector Laboratories - H3403) was used for the counterstaining of the sections, and slides were imaged using an Aperio ScanScope (Leica Biosystems, Buffalo Grove, IL, USA) with 2x - 40x objectives. Images were analyzed using Aperio ImageScope v12 software (Leica Biosystems, Buffalo Grove, IL, USA). Randomly selected 5 representative images at 40x magnification were used for quantification, and the 2 atrial chambers were analyzed separately. Quantified images were primarily obtained from the same section to ensure consistency. The percentage of SA-β-Gal-positive cells was calculated by manually counting SA-β-Gal-positive and SA-β-Gal-negative cells using the counter tool in ImageJ.

*Immunofluorescence (IF) staining*. 5 µm-thick frozen sections of rabbit or human atrial tissues were acquired and fixed in 4% ice cold PFA (in 1x PBS) for 10 minutes and then placed in fresh 1x PBS for 10 minutes. All steps were performed in a humidified chamber. The tissue was encircled with a hydrophobic pen and washed in PBS 3 times. For intracellular targets, frozen sections were permeabilized with 0.1% Triton X-100 in PBS for 30 min. Slides were blocked with 10% normal donkey or goat serum including Triton X-100 in PBS for 1 hr at room temperature. Slides were incubated with primary antibodies diluted in antibody dilution buffer prepared with BSA overnight at 4°C in the dark, then washed in PBS 3 times for 5 minutes each and incubated in suitable secondary antibodies diluted in antibody dilution buffer for 1 hr and 30 minutes in the dark at room temperature. Slides were again washed in PBS 3 times for 5 minutes each. When slides were being stained for multiple targets, the slides were placed in blocking buffer again and the steps of adding primary and secondary antibodies were repeated. Once all antibodies had been added, incubated, and washed off, slides were mounted with Prolong Gold Reagent with DAPI (Invitrogen, P36931). All antibodies used in the experiments were validated with rabbit and human atrial samples before initiating any experiments and are listed in **Supplemental Table 2**.

At least five random images with 10 Z-stacks were acquired per sample using a Nikon Ti2 confocal microscope with an A1R scanner at 60x magnification. Quantified images were primarily obtained from the same section to ensure consistency. Images were analyzed with Nikon NIS Element AR software and the number and percentage of cells with positive signals were derived from maximum intensity projection images by manually using the counter tool in ImageJ. Signal locations and cell morphologies were taken into consideration to determine positive signals. A γH2AX nuclear focus was defined as a distinct focus overlapping a DAPI-positive nucleus, while large, continuous signals within the γH2AX channel or any signal outside the nucleus were considered artifacts and excluded from analysis. As a general practice, nuclei with 3 or more γH2AX foci were counted as positive. Images co-stained for desmin and γH2AX were used to calculate overall γH2AX percentages.

| **Supplemental Table 2: Antibodies used in histology experiments** | | | |
| --- | --- | --- | --- |
| **Target** | **Host Species** | **Company** | **Product Number** |
| αSMA | Goat | Novusbio | NB300-978 |
| γH2AX (for rabbit tissue) | Mouse | Abcam | Ab26350 |
| γH2AX (for human tissue) | Rabbit | Cell Signaling | 2577S |
| Desmin | Goat | Invitrogen | PA5-19063 |
| CD31 | Goat | R&D Systems | AF3628 |
| CD68 | Rat | Invitrogen | 14-0681-82 |
| Mouse Alexa Fluor 594 | Goat | Invitrogen | A11005 |
| Rabbit Alexa Fluor 594 | Goat | Invitrogen | A110012 |
| Goat Alexa Fluor 647 | Donkey | Invitrogen | A21447 |
| Rat Alexa Fluor 647 | Goat | Invitrogen | A48265 |

*Immunohistochemistry (IHC) staining*. 6 µm sections of rabbit or human atrial tissues were obtained and fixed as described above. All steps were performed in a humidified chamber. The tissue was encircled with a hydrophobic pen and then washed in PBS 3 times. For intracellular targets, the slides were permeabilized using 0.1% Triton X-100 in PBS for 15 minutes. Slides were incubated in BLOXALL Blocking Solution (VECTOR Laboratories, SP-6000) for 10 minutes to quench endogenous peroxidase activity. The slides were then washed in PBS for 5 minutes. VECTASTAIN Elite ABC Kit was used for the assay (VECTOR Laboratories, VECTASTAIN Elite ABC Kit, Peroxidase (Mouse IgG), PK-6102). The slides were incubated in 1.5% blocking serum for 30 minutes at room temperature. After aspirating the blocking serum, the slides were incubated with a primary antibody (p16, Ventana, 705-4793) diluted as desired in 2.5% normal animal serum for 30 minutes, then washed in PBS for 5 minutes, and incubated with the secondary antibody diluted in 1.5% normal animal serum for 30 minutes at room temperature. After another 5-minute PBS wash, the slides were incubated in ABC solution (VECTASTAIN Elite ABC Reagent) for 30 minutes at room temperature. The slides were washed in PBS for 5 minutes and then incubated in DAB solution (VECTOR Laboratories, DAB Substrate Kit, Peroxidase (with Nickel), SK-4100) for 5-8 minutes before the slides were rinsed in tap water to stop the reaction and then rinsed in distilled water. Finally, the slides were counterstained with Nuclear Fast Red, dehydrated through a graded series of ethanol and xylene (1x 70% ethanol, 2x 100% ethanol, 3x Xylene), and mounted with Micromount medium (Leica). The slides were let dry overnight at room temperature and stored in a slide box. Slides were imaged using a Nikon Eclipse TE2000U Epifluorescence Microscope using a 60x Objective.

*Masson’s Trichrome Staining*. For fibrosis assessments, frozen sections of rabbit or human atrial tissues were stained with Masson’s Trichrome, and reagents from Electron Microscopy Sciences (catalog # 26367-series) were used. 10 µm sections were obtained and fixed as described above. Bouin’s Fixative was preheated to 56°C in a water bath. After briefly rinsing the slides in distilled water, the slides were placed in heated Bouin’s Fixative for 1 hour at 56°C in the water bath. The slides were washed until the yellow color of the fixative disappeared and then rinsed in distilled water before being stained in Weigert’s Iron Hematoxylin Working Solution (equal parts Weigert’s Iron Hematoxylin A and Hematoxylin B) for 5 minutes. The slides were washed in running tap water for 10 minutes, rinsed in distilled water, and then stained in Biebrich Scarlet-Acid Fuchsin for 15 minutes. After rinsing in distilled water, the slides were stained in Phosphomolybdic Acid-Phosphotungstic Acid for 15 minutes. The slides were then stained in Aniline Blue Solution for 15 minutes and rinsed in distilled water afterwards. Next, the slides were differentiated in 1% acetic acid for 4 minutes and then dehydrated through a graded series of ethanol and xylene (1x 70% ethanol, 2x 100% ethanol, 3x Xylene) before being mounted with Micromount medium (Leica).

Slides were imaged using an Aperio ScanScope (Leica Biosystems, Buffalo Grove, IL, USA) with a 2x - 40x objective. Randomly selected at least 5 representative images at 40x magnification were used for quantification, and the 2 atrial chambers were analyzed separately. Computerized assessment via Image J software and a script developed by Eric Mi (Koren laboratory) was used to calculate the percentage of fibrotic areas. All analyses were done blinded.

*RNA Isolation*. Total RNA was isolated from rabbit left and right atrial tissues using TRIzol (Invitrogen, 15596018) according to the manufacturer’s instructions. Nucleic acid concentration of the RNA was determined using a Nanodrop 2000c Spectrophotometer (Thermo Fisher, Waltham, MA, USA). Each sample was treated with the TURBO DNA-free Kit (Invitrogen, AM1907) according to the manufacturer’s instructions. RNA integrity was checked using an Agilent 2100 Bioanalyzer and Agilent (formerly AATI) Fragment Analyzer prior to use. As a general practice, samples with ≥8.5 RIN were used for downstream applications.

*RT-qPCR*. The iScript Reverse Transcription kit (BioRad, Hercules, CA, USA) and a C1000 Thermocycler (BioRad, Hercules, CA, USA) were used for the reverse transcription. For each gene of interest as well as an endogenous control, primer sets were created. Each gene's accession number was obtained from the National Center for Biotechnology Information gene database, and multiple primer sets were generated for each gene using the NIH’s Primer Blast tool. 100 ng, 10 ng, 1 ng, 0.1 ng, and 0.01 ng of cDNA from rabbit atrial tissue were used to calculate the effectiveness of each primer set. After further testing, suitable primer sets were chosen for each gene of interest based on their efficiencies (**Supplemental Table 3**). SYBR Green reagent (BioRad) and a ViiA 7 Real Time System (ThermoFisher, Waltham, MA, USA) were used to conduct RT-qPCR. SRP14 (internal control) and no template were used as controls. Normalized changes in gene expression were calculated using the ΔCT method. All experiments were run in triplicate for each primer set. Run results including CT values, standard curves, melt curves, and primer efficiency were exported by the ViiA7 QuantStudio software.

| **Supplemental Table 3: Rabbit-specific primers used in RT-qPCR experiments** | | |
| --- | --- | --- |
| **Target** | **Primer/probe Sequences (5’-3’)** | **Efficiency** |
| SRP14 | Accession: XM_002717984.2  Forward: 5’-TTCCAGAAATGCCGGTGTC  Reverse: 5’-GCTTCGATGCTGGACTTCTCA-3’ | 102% |
| p16 | Accession: XM_008255019.2  Forward: 5’-GCCGAAGGCGGTAACCA-3’  Reverse: 5’-CGCTCCCAGGCGAAGTT-3’ | 96% |
| p21 | Accession: XM_002714669.2  Forward: 5’-GAGACTGCGACGCACTCATG-3’  Reverse: 5’-CGCTCCCAGGCGAAGTT-3’ | 98% |

*Western Blots*. The 20-30mg of rabbit atrial tissue was rinsed in PBS, centrifuged, and homogenized in RIPA buffer (Boston BioProducts) containing a Protease/Phosphatase Inhibitor Cocktail (Cell Signaling Technology). After a sonication step, samples were rotated for 90 minutes at 4°C, centrifuged, and supernatants transferred to new tubes. Following protein quantification, 40–80 µg of heat-denatured (15 min at 60°C) protein was loaded for SDS-PAGE using 4x Laemmli Sample Buffer (Bio-Rad; final concentration: 2x Sample Buffer; 100 mM DTT). Protein was transferred to PVDF membranes, which were afterwards blocked in 3% BSA. Membranes were incubated with the primary antibodies overnight (**Supplemental Table 4**). Suitable secondary HRP-conjugated antibodies at 1:10,000 (Thermo Fisher) were used. SuperSignal West Pico PLUS Chemiluminescent Substrate (Thermo Fisher) and the ChemiDoc MP Imaging System (Bio-Rad) were used to detect signals. Protein band quantification was done with ImageLab software (Bio-Rad). Respective total protein expressions were normalized to GAPDH levels.

| **Supplemental Table 4: Antibodies used in WB experiments** | | | |
| --- | --- | --- | --- |
| **Target** | **Host Species** | **Company** | **Product Number** |
| p21 | Rabbit | Abcam | Ab109199 |
| p53 (total) | Mouse | Abcam | Ab17869-250 |
| Phospho-p53 (Ser 15) | Mouse | Cell Signaling | 9286S |
| GAPDH | Mouse | Thermo Fisher | 36-8600 |

*RNA-sequencing and analysis*. High quality RNA was isolated and sent to Azenta Life Sciences US, Inc. (Burlington, MA, USA) for RNA sequencing and library creation. Paired-end sequencing reads were quality-trimmed using fastp^5^ and aligned to the UM1 (UM_NZW_1.0) rabbit reference genome with STAR^6^. Gene-level counts were generated using featureCounts^7^ with the UM1 RefSeq transcript annotation (GCF_009806435.1). Count normalization was performed using the median-of-ratios method implemented in DESeq2^8^, followed by differential expression analysis using DESeq2’s negative binomial modeling framework. Normalized counts and differential expression results were further analyzed using custom R scripts. Gene set enrichment analysis (GSEA) was performed both in a targeted manner on the SenMayo gene set and in an unbiased manner using rabbit gene sets derived from Gene Ontology (GO) (obtained via the AnnotationHub R package^9^, accession AH107945) and KEGG (identifier T03373).

*Fisetin Preparation.* Fisetin (MedChemExpress, HY-N01892) was added at a concentration of 10 mg/mL to a solvent composed of 45% PEG300 (MedChemExpress, HY-Y0873), 5% Tween-80 (MedChemExpress, HY-Y1891), and 50% Saline. The solution was placed in a sonicator for 1 hour or until all the fisetin was completely dissolved.

*Aged Rabbit Fisetin Treatment Protocol.* Fisetin treatment was conducted on aged rabbits. The experimental group was given Fisetin in doses of 20 mg/kg, while the control group was given a vehicle (45% PEG300, 5% Tween-80, and 50% Saline). Both fisetin and vehicle were mixed with apple sauce and given to the rabbits orally via a syringe. All rabbits were assessed pre- and post-treatment via ECG and Echo recordings, and blood samples were collected prior to treatment, 8-12 hours after the first dose of each cycle, and prior to euthanizing the rabbits for beating heart harvest (BHH). The protocols were completed by the end of the 22nd day of treatment.

*Statistical Analysis*. Unless otherwise stated, all data are represented as mean ± SD in the text. To compare two groups, the two-tailed Exact test or Mann-Whitney U test was used. To compare more than two groups, ANOVA (Kruskal-Wallis test) with Dunn’s post-hoc test was applied. GraphPad Prism 9.5.1 was used to perform all statistics and create all graphs. Scatter plots show mean ± SEM, unless otherwise stated. All experiments and analysis were performed with the practice of randomization and blinding. The sample sizes in this study were chosen based on standard practices in the field and previous similar experiments reported by us^10^ and in the literature. The number of biological (N) and technical replicates was selected to ensure a representative assessment of the observed effects, while accounting for practical constraints such as tissue availability and experimental feasibility.

# Supplemental Figures

**Supplemental Figure 1: Fibrosis in the atria of aged and young rabbits**

**(A)** Representative M. Trichrome images of aged (4y6m) and young rabbit atria (11m). The scale bar of aged animal’s 2x image is 6mm. The sale bar of young animal’s 2x image is 4mm. Scale bars of 40x images are 60µm. **(B)** Quantification of percent fibrosis per chamber. Scatter plots = Mean±SEM, N=5 aged and N=7 young rabbits, Mann-Whitney U test.

**Supplemental Figure 2: Fibrosis in human RAA and LAA samples**

**(A**) Representative M. Trichrome images of RAA samples from patients with AF and SR. The scatter plot (Mean + SEM) on the right shows the percentage area of fibrosis of the RAA samples. **(B)** Representative M. Trichrome images of LAA samples (AF and SR). The scatter plot (Mean ± SEM) on the right indicates the percentage fibrosis of LAA samples. All scale bars are 10 μm. N=5 for AF, N=5 for SR, Mann-Whitney U test.

# References

1. Morrissey PJ, Murphy KR, Daley JM, Schofield L, Turan NN, Arunachalam K, et al. A novel method of standardized myocardial infarction in aged rabbits. *Am J Physiol Heart Circ Physiol*. 2017;312:H959-H967

2. Fedorov VV, Lozinsky IT, Sosunov EA, Anyukhovsky EP, Rosen MR, Balke CW, et al. Application of blebbistatin as an excitation-contraction uncoupler for electrophysiologic study of rat and rabbit hearts. *Heart Rhythm*. 2007;4:619-626

3. Ziv O, Morales E, Song YK, Peng X, Odening KE, Buxton AE, et al. Origin of complex behaviour of spatially discordant alternans in a transgenic rabbit model of type 2 long qt syndrome. *J Physiol*. 2009;587:4661-4680

4. Debacq-Chainiaux F, Erusalimsky JD, Campisi J, Toussaint O. Protocols to detect senescence-associated beta-galactosidase (sa-betagal) activity, a biomarker of senescent cells in culture and in vivo. *Nat Protoc*. 2009;4:1798-1806

5. Chen S, Zhou Y, Chen Y, Gu J. Fastp: An ultra-fast all-in-one fastq preprocessor. *Bioinformatics*. 2018;34:i884-i890

6. Dobin A, Davis CA, Schlesinger F, Drenkow J, Zaleski C, Jha S, et al. Star: Ultrafast universal rna-seq aligner. *Bioinformatics*. 2013;29:15-21

7. Liao Y, Smyth GK, Shi W. Featurecounts: An efficient general purpose program for assigning sequence reads to genomic features. *Bioinformatics*. 2014;30:923-930

8. Love MI, Huber W, Anders S. Moderated estimation of fold change and dispersion for rna-seq data with deseq2. *Genome Biol*. 2014;15:550

9. Morgan M, Shepherd L. Annotationhub: Client to access annotationhub resources. 2025

10. Baggett BC, Murphy KR, Sengun E, Mi E, Cao Y, Turan NN, et al. Myofibroblast senescence promotes arrhythmogenic remodeling in the aged infarcted rabbit heart. *Elife*. 2023;12
