# Supplementary material for: Senolytic reduction of senescent cells mitigates atrial arrhythmia vulnerability in aging rabbits
Source: Heart Rhythm. Author manuscript; Available in PMC 2026 Apr 13. (PMC13075518; doi:10.1016/j.hrthm.2026.01.007)
Supplement: Supplemental Figure 1 [file NIHMS2158865-supplement-Supplemental_Figure_1.pptx]

## Slide 1
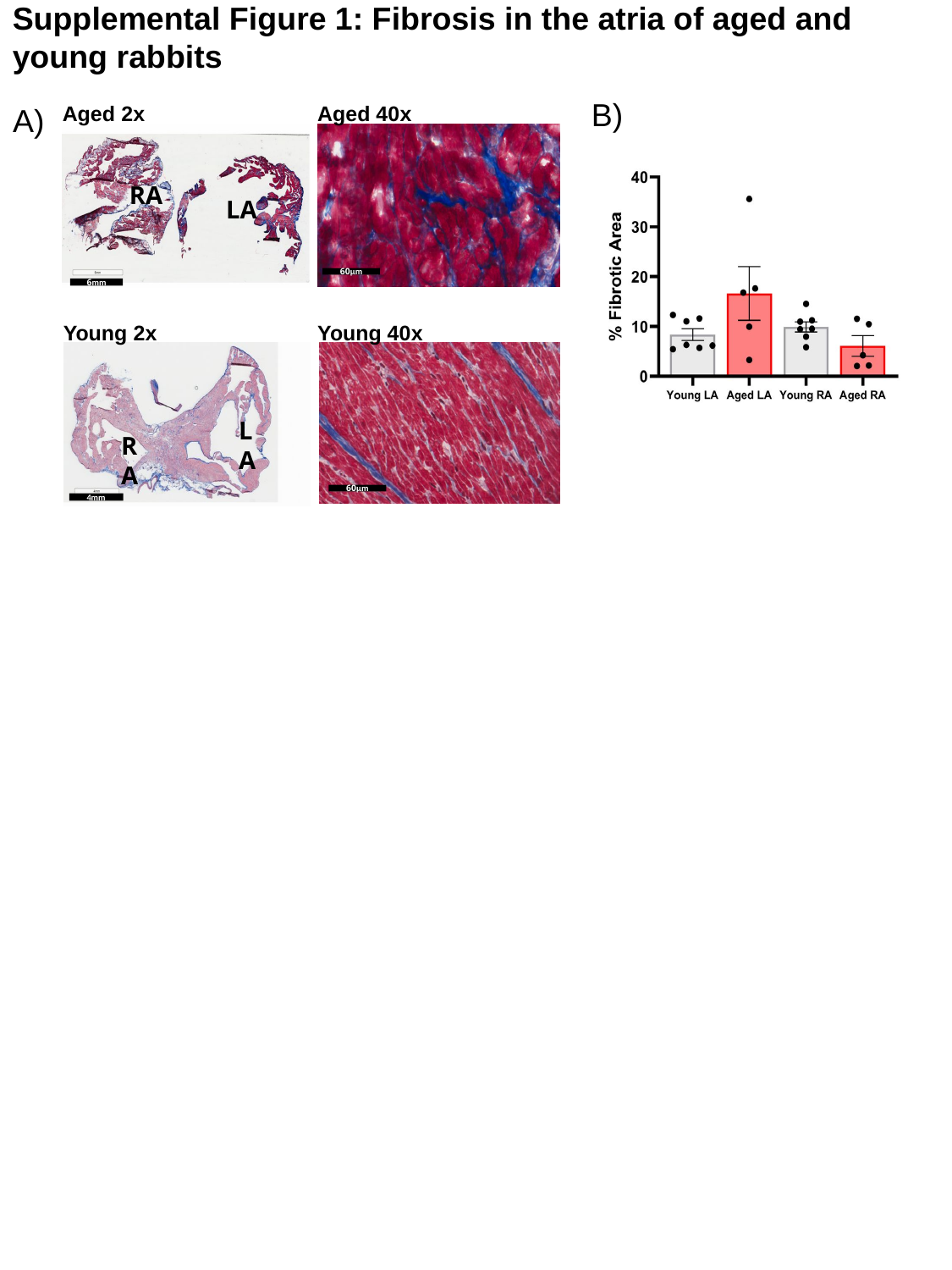

Supplemental Figure 1: Fibrosis in the atria of aged and young rabbits
Aged 40x
Aged 2x
A)
RA
LA
60μm
6mm
Young 40x
Young 2x
LA
RA
60μm
4mm
B)
