# Supplementary material for: Senolytic reduction of senescent cells mitigates atrial arrhythmia vulnerability in aging rabbits
Source: Heart Rhythm. Author manuscript; Available in PMC 2026 Apr 13. (PMC13075518; doi:10.1016/j.hrthm.2026.01.007)
Supplement: Supplemental Figure 2 [file NIHMS2158865-supplement-Supplemental_Figure_2.pptx]

## Slide 1
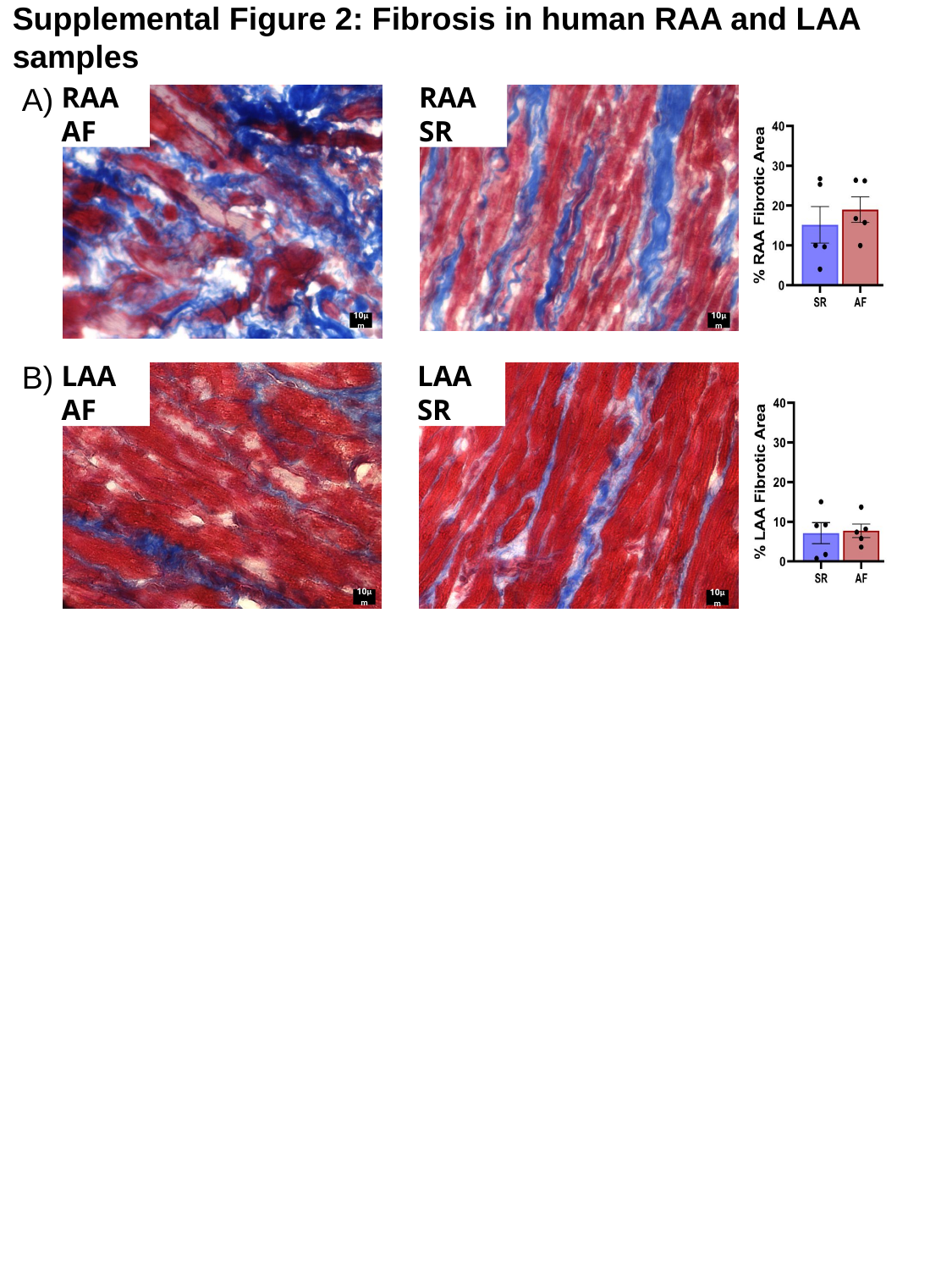

Supplemental Figure 2: Fibrosis in human RAA and LAA samples
A)
RAA AF
RAA SR
10μm
10μm
B)
LAA SR
LAA AF
10μm
10μm
